# Supplementary material for: A widely-occurring family of pore-forming effectors broadens the impact of the Serratia Type VI secretion system
Source: EMBO J. 2025 Oct 21;44(23):6892–918. doi: 10.1038/s44318-025-00587-x (PMC12669606; doi:10.1038/s44318-025-00587-x)
Supplement: Supplementary file 1 — Appendix [file 44318_2025_587_MOESM1_ESM.pdf]

Appendix

For: "A widely-occurring family of pore-forming effectors broadens the impact of the *Serratia* Type VI secretion system"

**Appendix Figure S1**	The structure of Sip4 predicted by AlphaFold2.
**Appendix Figure S2**	Removal of native cysteine residues and substitution of selected other residues with cysteine for mPEG-Mal topology mapping does not affect Sip4 functionality.
**Appendix Figure S3**	Purification and analysis of Ssp4.
**Appendix Figure S4**	No ion permeation is observed for a mock preparation of Ssp4 from *E. coli* cells without the vector directing expression of Ssp4.
**Appendix Figure S5**	Depiction of K^+ ions bound to a cluster of polar residues in the centre of the hydrated pore during molecular dynamics simulations of the $Ssp4_{114\text{-}302}$ monomer.
**Appendix Figure S6**	Validation of mutations identified by Tn-seq in *P. fluorescens* as potentially affecting susceptibility to Ssp4 or Ssp6.
**Appendix Figure S7**	Amino acid sequence alignment of identified Ssp4-like proteins.
**Appendix Table S1**	Summary of AlphaFold2 oligomer predictions for Ssp4 and stability of different oligomeric forms in a lipid bilayer according to molecular dynamics simulations.
**Appendix Table S2**	Strains and plasmids used in this study.
**Appendix Table S3**	Oligonucleotide primers used in this study.
**References**	

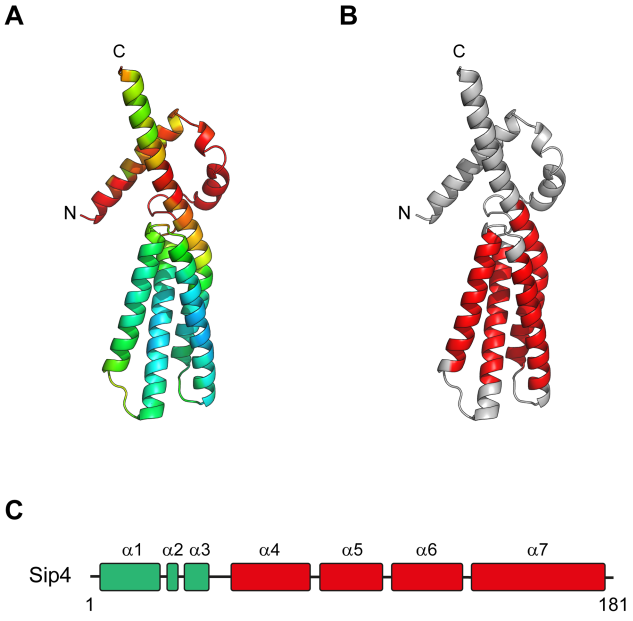

Appendix Figure S1. The structure of Sip4 predicted by AlphaFold2. (**A**) Structure coloured by pLDDT value, spectrum from red (<50), yellow, green, cyan, to blue (>90). (**B**) Regions predicted to form transmembrane helices by MEMSAT2 are highlighted in red (amino acids 53-74 in α4, amino acids 87-107 in α5, amino acids 114-135 in α6, and amino acids142-168 in α7). (**C**) Secondary structure elements in the predicted structure of Sip4, with α-helical regions predicted to include transmembrane helices coloured red.

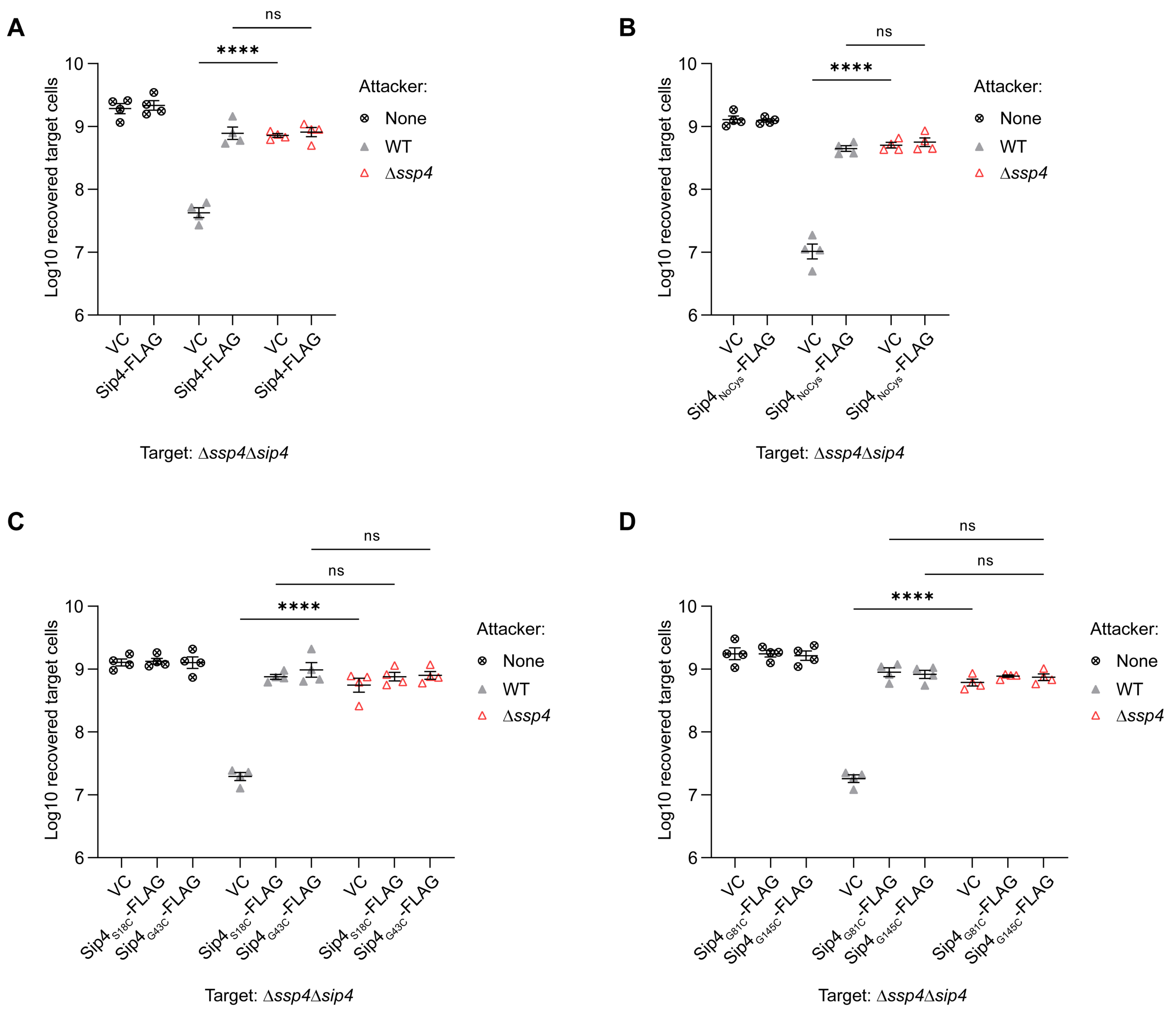

Appendix Figure S2. Removal of native cysteine residues and substitution of selected other residues with cysteine for mPEG-Mal topology mapping does not affect Sip4 functionality. Recovery of *S. marcescens* Db10 Δ*ssp4* Δ*sip4* target strains carrying the vector control (VC, pSUPROM) or plasmids directing the expression of (**A**) wild type Sip4 with C-terminal 3xFLAG tag (Sip4-FLAG), (**B**) a derivative lacking native Cys residues ($Sip4_{NoCys}$-FLAG, C60A-C127A-C128A), (**C**) derivatives of $Sip4_{NoCys}$-FLAG with S18C or G43C substitutions ($Sip4_{S18C}$-FLAG, $Sip4_{G43C}$-FLAG), or (**D**) derivatives of $Sip4_{NoCys}$-FLAG with G81C or G145C substitutions ($Sip4_{G81C}$-FLAG, $Sip4_{G145C}$-FLAG), following co-culture with wild type (WT) or Δ*ssp4* attacker strains of Db10. None, no attacker. Data are presented as mean +/- SEM with individual data points overlaid (n=4 biological replicates; **** p<0.0001, ns not significant, one-way ANOVA with Tukey's test; for clarity, only selected comparisons are displayed).

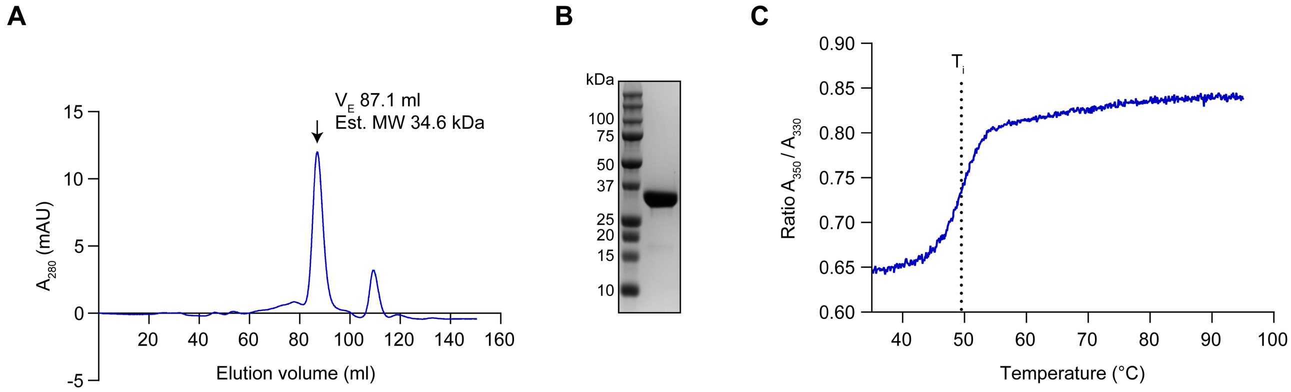

Appendix Figure S3. Purification and analysis of Ssp4. Overproduced His_6-GST-Ssp4 protein was purified to apparent homogeneity by Ni-NTA and GST-affinity chromatography. Following cleavage of His-GST, purified Ssp4 was separated by size exclusion chromatography (SEC). (**A**) SEC profile of purified Ssp4 separated using a Superdex 200 Hiload 26/600 column. The elution volume (V_E) and estimated molecular weight (Est. Mw) of the Ssp4 peak, based on calibration of the column with protein standards, is indicated. (**B**) Protein within the Ssp4 peak was visualised by SDS-PAGE and Coomassie staining. The purified Ssp4 protein has a predicted Mw of 35.5 kDa. (**C**) The unfolding profile of purified Ssp4 was determined by thermal calorimetry. The inflection temperature (Ti, 49.5°C) is displayed as a dotted line.

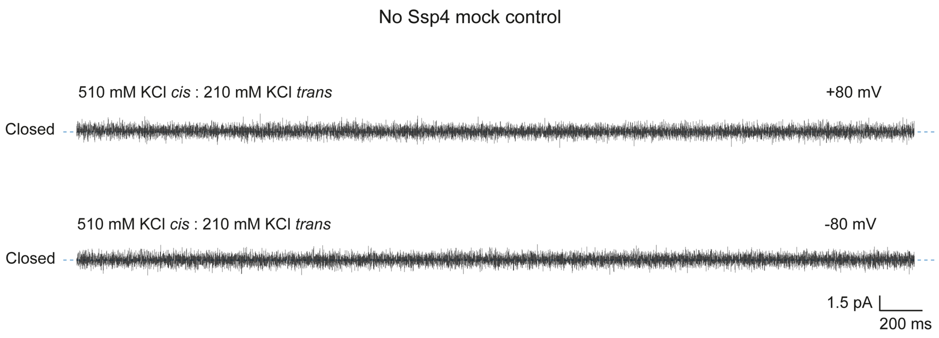

Appendix Figure S4. No ion permeation is observed for a mock preparation of Ssp4 from *E. coli* cells expressing GST but not Ssp4. Measurement of current fluctuations under voltage-clamp conditions under a holding command of +80 mV (top) or -80 mV (bottom) following addition of the mock protein preparation. The dotted line shows the baseline zero current. No current fluctuations were observed in three independent experiments.

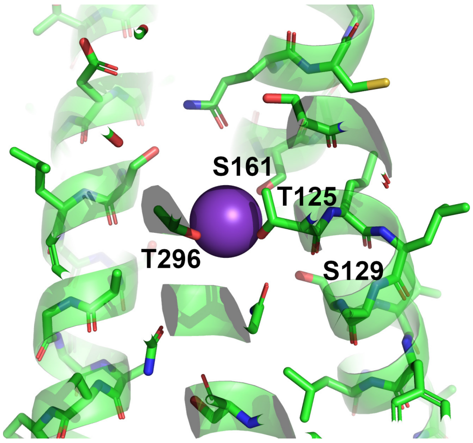

Appendix Figure S5. Depiction of K^+ ions bound to a cluster of polar residues in the centre of the hydrated pore during molecular dynamics simulations of the $Ssp4_{114-302}$ monomer.

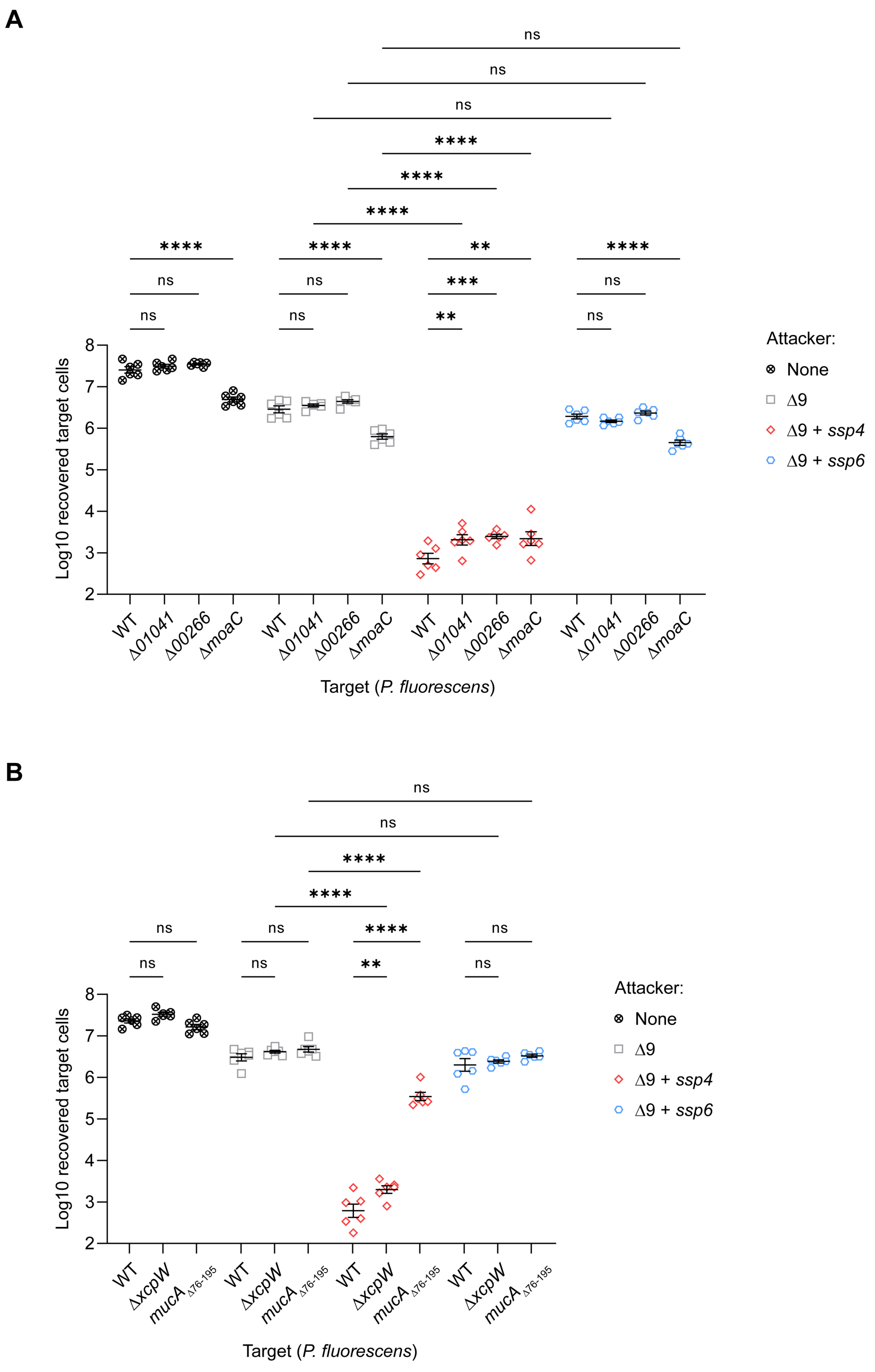

Appendix Figure S6. Validation of mutations identified by Tn-seq in *P. fluorescens* as potentially affecting susceptibility to Ssp4 or Ssp6. Recovery of wild type (WT) or reconstructed defined mutants (Δ*01041,* Δ*00266,* Δ*moaC*, panel **A**, or Δ*xcpW, mucA*$_{\Delta 76\text{-}195}$, panel **B**) of *P. fluorescens* 55, following co-culture with attacking strains of *S. marcescens* Db10 as indicated. None, no attacker. Data are presented as mean ± SEM with individual data points overlaid (n=6 biological replicates; **** P<0.0001, *** P<0.001, ** P<0.01, ns, not significant; one-way ANOVA with Tukey's test; for clarity, only selected comparisons are displayed). Panels A and B represent the full experiments from which Figure 7C and 7D, respectively, are taken.

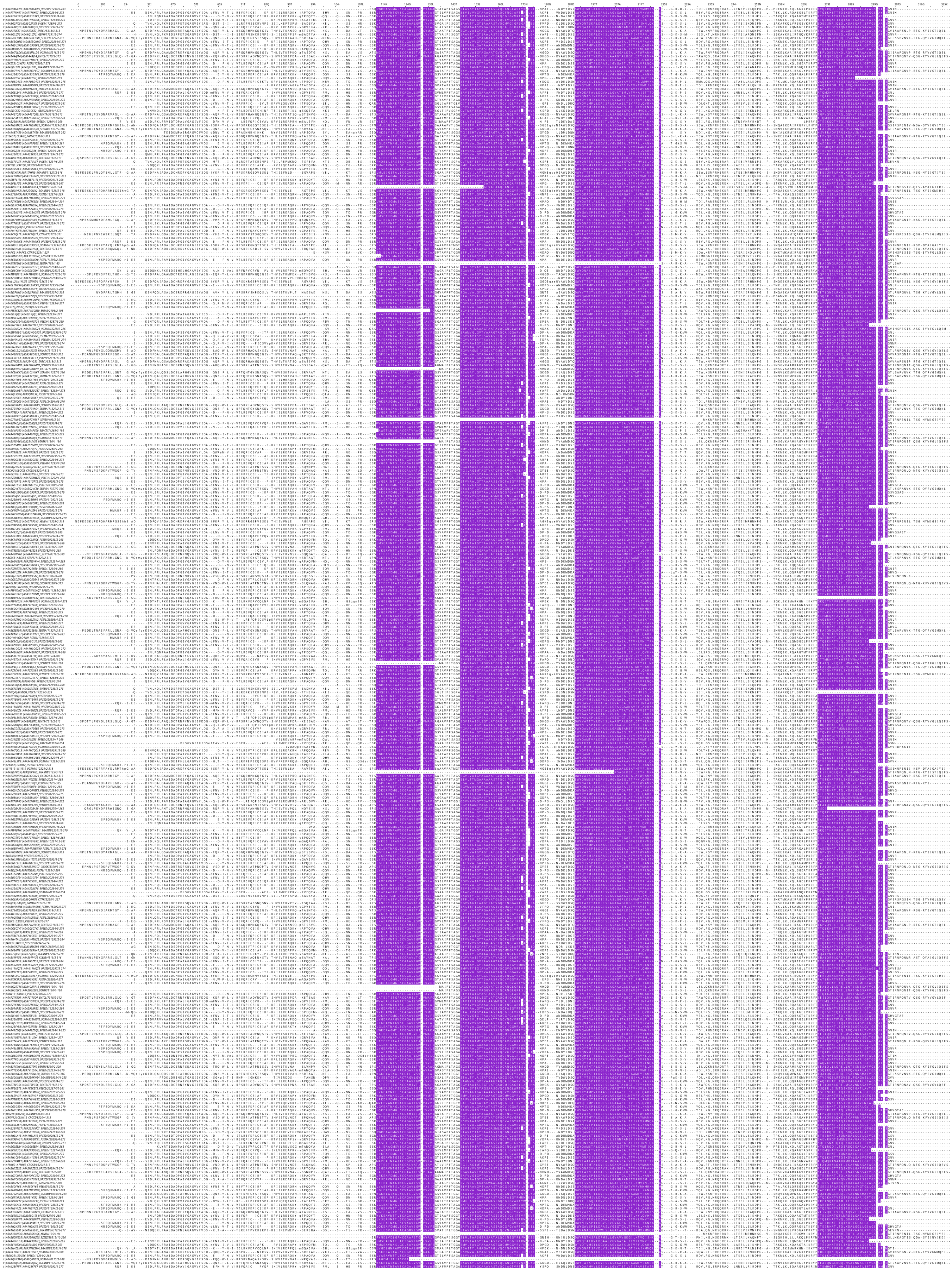

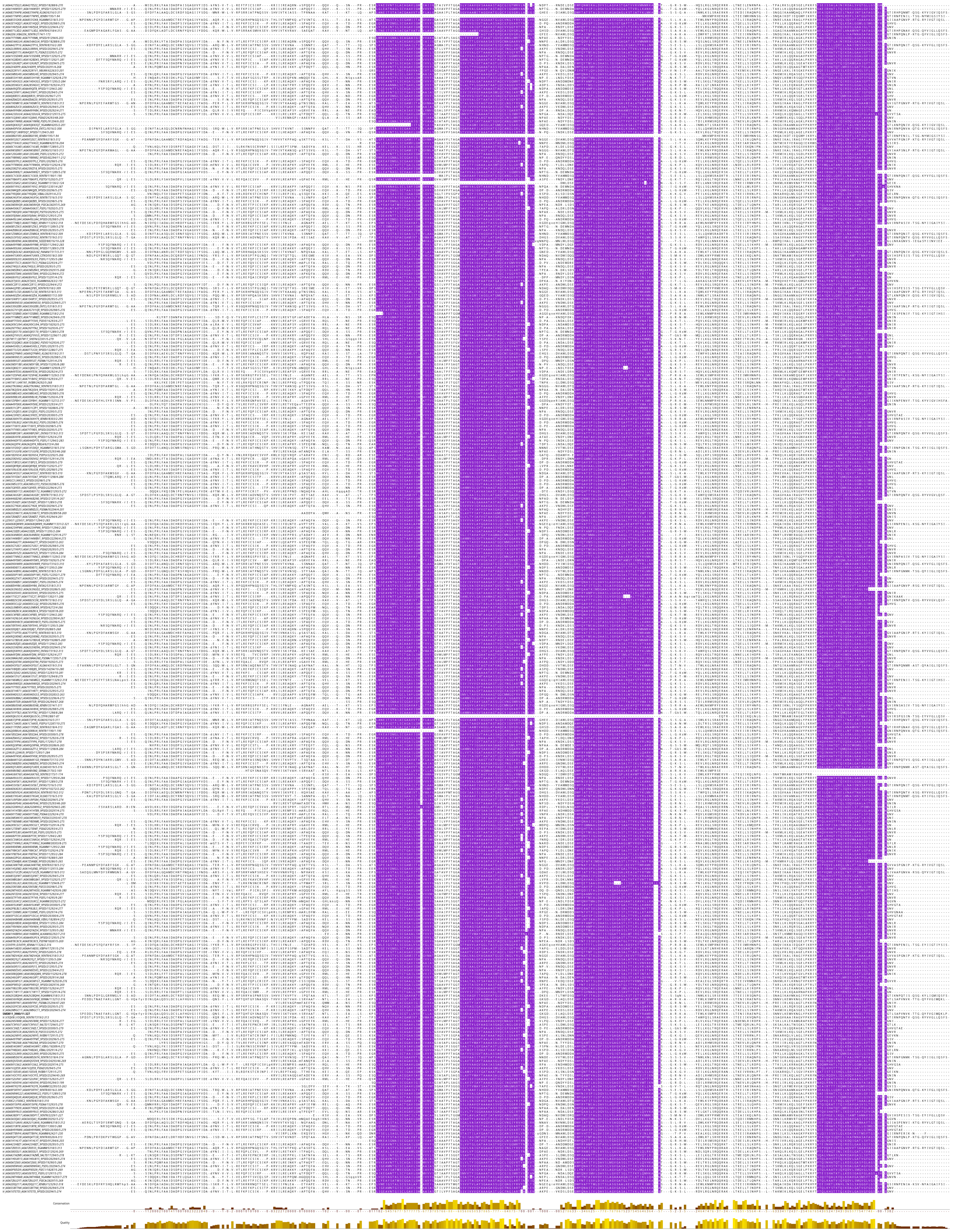
Conservation
Quality
Consensus

Appendix Figure S7. Amino acid sequence alignment of identified Ssp4-like proteins. The regions corresponding to the predicted transmembrane helices of *S. marcescens* Db10 Ssp4 (SMDB11_3980, bold) are coloured purple. Conservation, alignment quality and consensus sequence are shown below. Homologues are listed in Dataset EV2.

Appendix Table S1. Summary of AlphaFold2 oligomer predictions for Ssp4 and stability of different oligomeric forms in a lipid bilayer according to molecular dynamics simulations.

Oligomeric state of Ssp4	Average pLDDT score[1]	PAE plot[2]	Full length monomer-multimer monomer RMSD (Å)[3]	Truncated monomer-monomer backbone RMSD (Å)[4]	Average simulation RMSD (Å)[5]	Membrane disruption?
Monomer	63.9	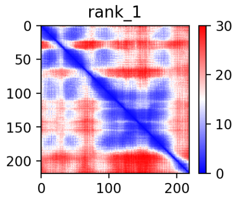	0.85 (168)	-	2.2	no
Dimer	56.1	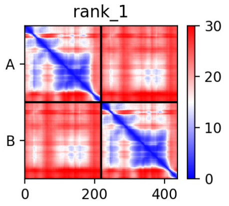	0.74 (164)	2.3	2.3	no
Trimer	56.1	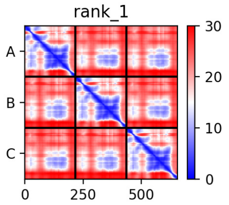	1.2 (180)	2.4	3.2	no
Tetramer	43.7	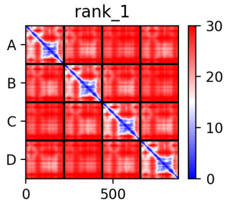	2.6 (180)	2.7	2.8	no
Pentamer	41.0	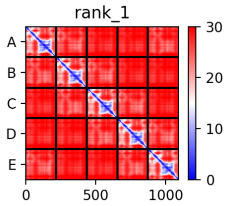	2.5 (172)	2.6	3.2	yes
Hexamer	45.0	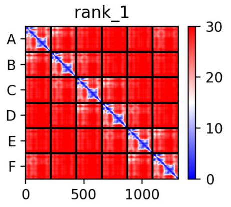	4.3 (192)	4.7	>5	yes

Heptamer	50.4	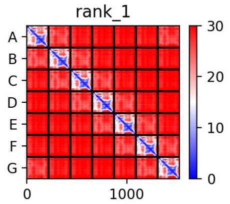	2.2 (198)	2.3	-	yes
Octamer	42.8	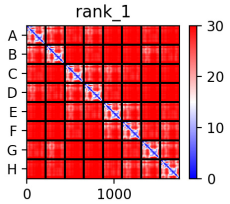	2.9 (190)	3.5	>5	yes

[1] According to AlphaFold2 prediction for the monomer or multimers of the truncated form $Ssp4_{114\text{-}331}$. pLDDT, predicted local distance difference test. Note that the average pLDDT value for the $Ssp4_{114\text{-}331}$ monomer is lower than for the full length monomer despite their highly similar structures. We interpret this as resulting from truncation of the multiple sequence alignment leading to lower AlphaFold2 confidence for predictions made using $Ssp4_{114\text{-}331}$.

[2] PAE (predicted aligned error) plots for AlphaFold2 structural predictions of the monomer or multimers of the truncated form $Ssp4_{114\text{-}331}$

[3] Comparison of the full length Ssp4 monomer ($Ssp4_{114\text{-}331}$) with individual monomers in the oligomeric assembly (predicted using $Ssp4_{114\text{-}331}$). Backbone Cα RMSD values were determined using Pymol with the number of aligned amino acids over which the value was calculated is noted in brackets.

[4] Comparison of the truncated Ssp4 monomer ($Ssp4_{114\text{-}302}$) with individual monomers in the oligomeric assembly (containing $Ssp4_{114\text{-}302}$). Backbone Cα RMSD values were calculated over the length of the protein using GROMACS.

[5] Assessment of stability of the assembly in the model membrane, with value >5 indicating instability

Appendix Table S2. Strains and plasmids used in this study.

Name	Description	Reference / Source
**<u>Bacterial strains</u>**		
Serratia marcescens		
Db10	Wild type strain	Flyg *et al*, 1980
SJC11	Db10 Δ*tssE* (Δ*SMDB11_2271*)	Murdoch *et al*, 2011
MJF8	Db10 Δ*ssp4* (Δ*SMDB11_3980*)	Fritsch *et al*, 2013
JAD01	Db10 Δ*ssp4*Δ*sip4* (Δ*SMDB11_3980-3979*)	Fritsch *et al*, 2013
JAD06/AO01	Db10 Δ*ssp4*Δ*sip4* (Δ*SMDB11_3980-3979*), Str^R	Fritsch *et al*, 2013
AO03	Db10 Δ*ssp4*Δ*sip4* Δ*lacZ*::P$_{T5}$-*gfpmut2* kan^R. Encodes cytoplasmic GFP, IPTG-inducible and expressed constitutively at a low level.	This study
AO07	Db10 Δ*lacZ*::P$_{T5}$-*mCherry* kan^R. Encodes cytoplasmic mCherry, IPTG-inducible and expressed constitutively at a low level.	Mariano *et al*, 2019
AO08	Db10 Δ*tssE* Δ*lacZ*::P$_{T5}$-*mCherry* kan^R	Mariano *et al*, 2019
AO09	Db10 Δ*ssp4* Δ*lacZ*::P$_{T5}$-*mCherry* kan^R	This study
YL37	Db10 Δ9 [Δ*ssp1* (Δ*SMDB11_2261*), Δ*ssp2* (Δ*SMDB11_2264*), Δ*ssp3*/*tfe1* (*ΔSMDB11_1112*), Δ*ssp4* (Δ*SMDB11_3980*), Δ*ssp5* (*ΔSMDB11_4628*), Δ*ssp6* (Δ*SMDB11_4673*), Δ*rhs1* (Δ*SMDB11_2278*), *rhs2*$_{H1369A}$ (*SMDB11_1610*$_{H1369A}$), Δ*slp* (Δ*SMDB11_0927*)]	This study; constituent mutations in Alcoforado Diniz & Coulthurst, 2015; Cianfanelli *et al*, 2016; Trunk *et al*, 2018
YL57	Db10 Δ9 + *ssp4* (Δ*ssp1* Δ*ssp2* Δ*ssp3*/*tfe* Δ*ssp5* Δ*ssp6* Δ*rhs1* *rhs2*$_{H1369A}$ Δ*slp*); generated by restoring the wild type *ssp4* allele in YL37	This study
GM103	Db10 Δ9 + *ssp6* (Δ*ssp1* Δ*ssp2* Δ*ssp3*/*tfe1* Δ*ssp4* Δ*ssp5* Δ*rhs1* *rhs2*$_{H1369A}$ Δ*slp*); generated by restoring the wild type *ssp6* allele in YL37	This study
YL56	Db10 Δ9 + *ssp2* (Δ*ssp1* Δ*ssp3*/*tfe1* Δ*ssp4* Δ*ssp5* Δ*ssp6* Δ*rhs1* *rhs2*$_{H1369A}$ Δ*slp*); generated by restoring the wild type *ssp2* allele in YL37	This study
YL33	Db10 Δ9 + *rhs2* (Δ*ssp1* Δ*ssp2* Δ*ssp3*/*tfe1* Δ*ssp4* Δ*ssp5* Δ*ssp6* Δ*rhs1* Δ*slp*)	This study; constituent mutations in Alcoforado Diniz & Coulthurst, 2015; Cianfanelli *et al*, 2016; Trunk *et al*, 2018
Pseudomonas fluorescens		
P. fluorescens 55	Wild type strain	Murdoch *et al*, 2011
55 (mScarlet)	*P. fluorescens 55* P$_{rpsG}$-mScarlet Gen^R	This study
MR13 (mScarlet)	*P. fluorescens 55* Δ*01041* (Δ*33931E_Pfluorescens55_01041*), P$_{rpsG}$-mScarlet Gen^R	This study
MR14 (mScarlet)	*P. fluorescens 55* Δ*00266* (Δ*33931E_Pfluorescens55_00266*), P$_{rpsG}$-mScarlet Gen^R	This study
MR15 (mScarlet)	*P. fluorescens 55* Δ*moaC* (Δ*33931E_Pfluorescens55_01260*), P$_{rpsG}$-mScarlet Gen^R	This study
MR16 (mScarlet)	*P. fluorescens 55* Δ*xcpW* (Δ*33931E_Pfluorescens55_02495*), P$_{rpsG}$-mScarlet Gen^R	This study
MR17 (mScarlet)	*P. fluorescens 55* *mucA*$_{Δ76\text{-}195}$ (*33931E_Pfluorescens55_01704*$_{Δ76\text{-}195}$), encodes MucA lacking C-terminal 120 amino acids; P$_{rpsG}$-mScarlet Gen^R	This study
MR28 (mScarlet)	*P. fluorescens 55* Δ*algD* (Δ*33931E_Pfluorescens55_01256*), P$_{rpsG}$-mScarlet Gen^R	This study
MR29 (mScarlet)	*P. fluorescens 55* *mucA*$_{Δ76\text{-}195}$ Δ*algD*, P$_{rpsG}$-mScarlet Gen^R	This study
MR18	*P. fluorescens 55* *att*Tn*7*::P$_{RhaBAD}$-*sp-ssp4*, Gen^R	This study
MR19	*P. fluorescens 55* *att*Tn*7*::P$_{RhaBAD}$-*sp-ssp6*, Gen^R	This study
MR20	*P. fluorescens 55* *att*Tn*7*::P$_{RhaBAD}$, Gen^R	This study

Escherichia coli		
MG1655	Wild type (model K-12 strain)	Blattner *et al*, 1997
CC118λ*pir*	Donor strain for pKNG101-derived allelic exchange plasmids (λ*pir*)	Herrero *et al*, 1990
HH26 pNJ5000	Helper strain for mobilization of pKNG101	Grinter, 1983
SM10λpir	Donor strain for conjugal transfer of pMQ30, pTNS1 and pIT2	Choi & Schweizer, 2006b
HB101 pRK2013	Helper strain for mobilization of pUC18T-miniTn*7*T-Gen^R and pJM220	Choi & Schweizer, 2006b
BL21(DE3)	Protein overexpression strain. Chromosomal λDE3 encodes IPTG-inducible T7 RNA polymerase.	Novagen
sHuffle T7	*E. coli* K-12 expressing DsbC to promote disulfide bond formation.	NEB
BW25113	Parental strain of the Keio collection. Δ(*araD-araB*)*567*, Δ*lacZ4787 (::rrnB-4), lacIp-4000(lacI^Q), λ$^-$, rpoS369(Am), rph-1,* Δ*(rhaD-rhaB)568, hsdR514*	Baba *et al*, 2006
-	BW25113 Δ*lacA*::Kan^R (*b0342*) from Keio collection	Baba *et al*, 2006
-	BW25113 Δ*cpxA*::Kan^R (*b3911*) from Keio collection	Baba *et al*, 2006
-	BW25113 Δ*cpxR*::Kan^R (*b3912*) from Keio collection	Baba *et al*, 2006
MR21	BW25113 Δ*lacA*	This study
MR22	BW25113 Δ*cpxA*	This study
MR23	BW25113 Δ*cpxR*	This study
Enterobacter cloacae		
KM39	*Enterobacter cloacae* ATCC14037 Δ*tssE* (ΔECL_01548)	Lab collection
Burkholderia thailandensis		
tnbt1_er100415p08q104	*Burkholderia thailandensis* E264 with T23 transposon insertion in BTH_I2967	Gallagher *et al*, 2013
**<u>Plasmids</u>**		
**Mutant generation**		
pKNG101	Suicide vector for allelic exchange in *S. marcescens* (Str^R, *sacBR, mobRK2, oriR6K*)	Kaniga *et al*, 1991
pSAN72	pKNG101-derived allelic exchange plasmid for the generation of chromosomal Δ*lacZ*::P$_{T5}$-*gfpmut2 kan^R*	Gerc *et al*, 2015
pSC1706	pKNG101-derived allelic exchange plasmid for the generation of chromosomal Δ*lacZ*::P$_{T5}$-*mCherry kan^R*	Mariano *et al*, 2019
pSC1829	pKNG101-derived allelic exchange plasmid for restoration of the wild type *ssp2* allele in YL37	This study
pSC1830	pKNG101-derived allelic exchange plasmid for restoration of the wild type *ssp4* allele in YL37	This study
pSC2526	pKNG101-derived allelic exchange plasmid for restoration of the wild type *ssp6* allele in YL37	This study
pMQ30	Suicide vector for allelic exchange in *P. fluorescens* (Amp^R, *sacBR, mobIncP, oriColE1*)	Shanks *et al*, 2006
pSC3443	pMQ30-derived allelic exchange plasmid for the generation of chromosomal in-frame Δ*33931E_Pfluorescens55_01041* deletion	This study
pSC3444	pMQ30-derived allelic exchange plasmid for the generation of chromosomal in-frame Δ*33931E_Pfluorescens55_00266* deletion	This study
pSC3445	pMQ30-derived allelic exchange plasmid for the generation of chromosomal in-frame Δ*33931E_Pfluorescens55_01260*	This study
pSC3447	pMQ30-derived allelic exchange plasmid for the generation of chromosomal in-frame Δ*33931E_Pfluorescens55_02495* deletion	This study
pSC3454	pMQ30-derived allelic exchange plasmid for the generation of chromosomal in-frame deletion of amino acids 76-195 in MucA (33931E_Pfluorescens55_01704)	This study

pSC3464	pMQ30-derived allelic exchange plasmid for the generation of chromosomal in-frame Δ*33931E_Pfluorescens55_01256* deletion	This study
pUC18T-miniTn*7*T-Gm^R P$_{rpsG}$-mScarlet	Suicide vector for integration of gentamycin resistance cassette and constitutively expressed mScarlet into *att*Tn*7* site of *P. fluorescens* (Gen^R, Amp^R, *oriPMB1*)	Babin *et al*, 2016
pTNS1	Plasmid directing Tn*7* transposase expression for Tn*7* integration in *P. fluorescens*	Choi & Schweizer, 2006a
pJM220	Suicide vector for integration of gentamycin resistance cassette and gene of interest under the control of rhamnose-inducible promoter in the *att*Tn*7* site of *P. fluorescens* (Gen^R, Amp^R, *oriPMB1*, *rhaSR*-P$_{RhaBAD}$).	Meisner & Goldberg, 2016
pSC3457	Coding sequence for a fusion of the N-terminal signal peptide from *E. coli* OmpA to Ssp4 (sp-Ssp4) in pJM220	This study
pSC3459	Coding sequence for a fusion of the N-terminal signal peptide from *E. coli* OmpA to Ssp6 (sp-Ssp4) in pJM220	This study
pCP20	Temperature-sensitive plasmid for thermal induction of FLP recombinase (Amp^R, Cml^R, *ori pSC101*)	Cherepanov & Wackernagel, 1995
pIT2	Vector containing Tn*5*-based transposon T8 (IS*lacZ*/hah-*tet*) for generation of transposon library in *P. fluorescens*	Jacobs *et al*, 2003
**Heterologous gene expression in *E. coli***		
pBAD18-Kn	Arabinose-inducible expression vector; gene of interest is cloned downstream of the P$_{ara}$ promoter (Kan^R)	Guzman *et al*, 1995
pSC1234	Coding sequence for a fusion of the N-terminal signal peptide from *E. coli* OmpA to Ssp4 (sp-Ssp4) in pBAD18-Kn	Fritsch *et al*, 2013
pSC861	Coding sequence for sp-Ssp4 + Sip4 in pBAD18-Kn	Fritsch *et al*, 2013
pSC1236	Coding sequence for a fusion of the N-terminal signal peptide from *E. coli* OmpA to Ssp6 (sp-Ssp6) in in pBAD18-Kn	Fritsch *et al*, 2013
pSC1271	Coding sequence for sp-Ssp6 + Sip6 in pBAD18-Kn	Mariano *et al*, 2019
pSC838	Coding sequence for Ssp5 in pBAD18-Kn	Fritsch *et al*, 2013
pSC839	Coding sequence for Ssp5 + Sip5a in pBAD18-Kn	Fritsch *et al*, 2013
**Protein production**		
pHis-GEX-6P-1	Protein overexpression vector for fusion with PreScission-cleavable N-terminal His$_6$/GST tag under the control of the Tac promoter. Derived from pGEX-6P-1 (Amp^R)	van Aalten lab
pSC3407	Coding sequence for Ssp4 in pHis-GEX-6P-1, for production of PreScission-cleavable N-terminal His$_6$-GST-tagged Ssp4	This study
**Gene expression *in trans* in *S. marcescens***		
pSUPROM	Vector for constitutive expression of cloned genes under the control of the *E. coli tat* promoter (Kan^R)	Jack *et al*, 2004
pSC2305	Coding sequence for Sip4 with a C-terminal 3xFLAG tag (Sip4-FLAG) in pSUPROM	This study
pSC2310	Coding sequence for Sip4 with a C-terminal 3xFLAG tag and containing C60A, C127A and C128A substitutions (Sip4$_{NoCys}$-FLAG) in pSUPROM	This study
pSC2315	pSC2310 with S18C substitution in Sip4$_{NoCys}$-FLAG	This study
pSC2316	pSC2310 with G43C substitution in Sip4$_{NoCys}$-FLAG	This study
pSC2320	pSC2310 with G81C substitution in Sip4$_{NoCys}$-FLAG	This study
pSC2321	pSC2310 with G145C substitution in Sip4$_{NoCys}$-FLAG	This study

Appendix Table S3. Oligonucleotide primers used in this study.

Plasmid	Sequence of relevant primers (5'-3')	Description
pSC1829	TATATCTAGACCACACTTGCAATTCCTGC	Forward primer to clone *ssp2* and flanking regions into pKNG101
	TATAGGGCCCTTGTTCAAATGCATCCATCG	Reverse primer to clone *ssp2* and flanking regions into pKNG101
pSC1830	TATATCTAGAAGCTTCCTCAAGTTCTGCCAAC	Forward primer to clone *ssp4* and flanking regions into pKNG101
	TATAGGGCCCAAGTTTTTCCGTTCTGACGGTTTATC	Reverse primer to clone *ssp4* and flanking regions into pKNG101
pSC2526	TATATCTAGAGGGATCAGTTCGATGTGCG	Forward primer to clone *ssp6* and flanking regions into pKNG101
	TATAGGGCCCCGACGACATCAAGTACCTGTTCG	Reverse primer to clone *ssp6* and flanking regions into pKNG101
pSC3443	ATATCTAGAGAATGTGGAGTGGTGGCAC	Upstream forward primer for 01041 deletion in *P. fluorescens 55*
	ATAGGATCCCATCAGATACTCTCCCGTTC	Upstream reverse primer for 01041 deletion in *P. fluorescens 55*
	ATAGGATCCGAGCCAGCTTCGAATTGACAT	Downstream forward primer for 01041 deletion in *P. fluorescens 55*
	ATAGAATTCCGGGCAAGTCACGTATGTG	Downstream reverse primer for 01041 deletion in *P. fluorescens 55*
pSC3444	ATATCTAGACCTGGATTCGCATCTGCG	Upstream forward primer for 00266 deletion in *P. fluorescens 55*
	ATAGTCGACAGTCATGCTGCTGTTCTCC	Upstream reverse primer for 00266 deletion in *P. fluorescens 55*
	ATAGTCGACGTGTTCTAGGCACCAAATGG	Downstream forward primer for 00266 deletion in *P. fluorescens 55*
	ATAGAATTCTAATCGGCATGCCGCGTC	Downstream reverse primer for 00266 deletion in *P. fluorescens 55*
pSC3445	ATATCTAGACAGGAAATCGGCTTCCTGC	Upstream forward primer for *moaC* deletion in *P. fluorescens 55*
	ATAGGATCCATCGAGATGAGTCAGCACG	Upstream reverse primer for *moaC* deletion in *P. fluorescens 55*
	ATAGGATCCGCATGAGCATCAACGTATTGT	Downstream forward primer for *moaC* deletion in *P. fluorescens 55*
	ATAGAATTCCGCGTCTTCAAGTAATCCATC	Downstream reverse primer for *moaC* deletion in *P. fluorescens 55*
pSC3447	ATATCTAGAGGTGAAACGATGGGCGAATG	Upstream forward primer for *xcpW* deletion in *P. fluorescens 55*
	ATAGGATCCCTGATTCATGGCAGGCGC	Upstream reverse primer for *xcpW* deletion in *P. fluorescens 55*
	ATAGGATCCCCGTTGAACTAACTCAACAACAC	Downstream forward primer for *xcpW* deletion in *P. fluorescens 55*
	ATAGAATTCGAGCAACGCCAAGTGGTG	Downstream reverse primer for *xcpW* deletion in *P. fluorescens 55*
pSC3464	AAATCTAGAGCAAGTGCTATCAATATAGTACC	Upstream forward primer for *algD* deletion in *P. fluorescens 55*
	AAAGGATCCCATCGCATTTACCTCTTTGTC	Upstream reverse primer for *algD* deletion in *P. fluorescens 55*
	AAAGGATCCGGTATTTGCTGGTAAGAGC	Downstream forward primer for *algD* deletion in *P. fluorescens 55*
	AAAGAATTCGGAAACCGAAGGCCAG	Downstream reverse primer for *algD* deletion in *P. fluorescens 55*
pSC3454	ATATCTAGAGGTGATAGCGCGTTTTACAC	Upstream forward primer for deletion of part of *mucA* gene encoding MucA amino acids 76–195 in *P. fluorescens 55*
	ATAGTCGACGGCATCACGACTCATGGC	Upstream reverse primer for deletion of part of *mucA* gene encoding MucA amino acids 76–195 in *P. fluorescens 55*
	ATAGTCGACTGCCGGAACGGCTTCATC	Downstream forward primer for deletion of part of *mucA* gene encoding MucA amino acids 76–195 in *P. fluorescens 55*
	ATAGAATTCTGCAGATCGCGATCATCCG	Downstream reverse primer for deletion of part of *mucA* gene encoding MucA amino acids 76–195 in *P. fluorescens 55*

pSC3457	TTTACTAGTGCTAGCGAATTCGAGCTC	Forward primer to amplify sequence encoding sp-Ssp4 for cloning into pJM220
	TTTGGGCCCTACCTCACCATCTGCGG	Reverse primer to amplify sequence encoding sp-Ssp4 for cloning into pJM220<br>(final insert sequence below)
pSC3459	TTTACTAGTGCTAGCGAATTCGAGCTC	Forward primer to amplify sequence encoding sp-Ssp6 for cloning into pJM220
	TTTGGGCCCTTTCGACACCTTTTCAAAAAATAG	Reverse primer to amplify sequence encoding sp-Ssp6 from for cloning into pJM220
	ATAGCCCGGTACCTCGCGAAGGCCT	Forward primer for site-directed mutagenesis for incorporation of stop codon at the end of sp-Ssp6
	TTCGACACCTTTTCAAAAAATAGCTCGGTAATG CG	Reverse primer for site-directed mutagenesis for incorporation of stop codon at the end of sp-Ssp6<br>(final insert sequence below)
pSC3407	GCATGGATCCATGAAAACTGCTTTTTCTACGCC	Forward primer to amplify the sequence encoding Ssp4 for cloning into pHis-GEX-6P-1
	GCATGTCGACTTACCTCACCATCTGCGG	Reverse primer to amplify the sequence encoding Ssp4 for cloning into pHis-GEX-6P-1
pSC3460	TCTGTTCCAGGGGCCCCTGGCAAAGGTGCGA AGGAAATC	Forward primer for *ssp6* cloning into BamHI/SalI digested pSC3407 by Gibson assembly
	TCACGATGCGGCCGCTCGAGCTATTTCGACACC TTTTCAAAAAATAG	Reverse primer for *ssp6* cloning into BamHI/SalI digested pSC3407 by Gibson assembly
pSC2305	TATAGGATCCGTAATGGCGGAGTGGATGATTA AAC	Forward primer to amplify sequence encoding Sip4-FLAG for cloning into pSUPROM (template sequence below)
	TATAGTCGACCGGTTTACTTGTCATCGTCATCC	Reverse primer to amplify sequence encoding Sip4-FLAG for cloning into pSUPROM (template sequence below)
pSC2310	GCATGCTCGGTCTGGCTGCCCTCGGCGCCGC	Forward primer for introduction of C127A and C128A mutations into Sip4-FLAG encoded on pSC2305 by QuikChange site-directed mutagenesis
	GCGGCGCCGAGGGCAGCCAGACCGAGCATGC	Reverse primer for introduction of C127A and C128A mutations into Sip4-FLAG encoded on pSC2305 by QuikChange site-directed mutagenesis
	TATAGGATCCGTAATGGCGG	Forward primer 1 for introduction of C60A mutation into Sip4-FLAG encoded on pSC2305 by overlap PCR
	CGTCAGGGCCACCATC	Reverse primer 1 for introduction of C60A mutation into Sip4-FLAG encoded on pSC2305 by overlap PCR
	GATGGTGGCCCTGACG	Forward primer 2 for introduction of C60A mutation into Sip4-FLAG encoded on pSC2305 by overlap PCR
	TATAGTCGACCGGTTTACTTGTCATC	Reverse primer 2 for introduction of C60A mutation into Sip4-FLAG encoded on pSC2305 by overlap PCR
pSC2315	TATAGAATTCTGTCGGTTGGCG	Forward primer 1 for introduction of S18C mutation into Sip4-FLAG encoded on pSC2310 by overlap PCR
	CGATTTGGCATTCCATCTC	Reverse primer 1 for introduction of S18C mutation into Sip4-FLAG encoded on pSC2310 by overlap PCR
	GAGATGGAATGCCAAATCG	Forward primer 2 for introduction of S18C mutation into Sip4-FLAG encoded on pSC2310 by overlap PCR (used with pSC2310 Reverse primer 2)
pSC2316	CGCTGGCACGGCGTC	Reverse primer 1 for introduction of G43C mutation into Sip4-FLAG encoded on pSC2310 by overlap PCR (used with pSC2315 Forward primer 1)
	GACGCCGTGCCAGCG	Forward primer 2 for introduction of G43C mutation into Sip4-FLAG encoded on pSC2310 by overlap PCR (used with pSC2310 Reverse primer 2)
pSC2320	GGTCTTGCAGCTCCAGG	Reverse primer 1 for introduction of G81C mutation into Sip4-FLAG encoded on pSC2310 by overlap PCR (used with pSC2315 Forward primer 1)
	CCTGGAGCTGCAAGACC	Forward primer 2 for introduction of G81C mutation into Sip4-FLAG encoded on pSC2310 by overlap PCR (used with pSC2310 Reverse primer 2)
pSC2321	AGGGCGCAGGTGGCG	Reverse primer 1 for introduction of G145C mutation into Sip4-FLAG encoded on pSC2310 by overlap PCR (used with pSC2315 Forward primer 1)
	CGCCACCTGCGCCCT	Forward primer 2 for introduction of G145C mutation into Sip4-FLAG encoded on pSC2310 by overlap PCR (used with pSC2310 Reverse primer 2)

Plasmid	Template / final insert sequences
pSC2305	GTAATGGCGGAGTGGATGATTAAACGCCTGATCGTGCAGGGTCGGGAGATGGAAAGCCAAATCGCGGC<br>GCGCGATCGCGCACTTTTCGCCGGTGTGGATGCGCAGCTCGAGCAGCATTTCATGACGCCGGGCCAGCG<br>GTTGTCGCCGCCGGGCGTCGGCAACGTCATGTTGCTGATGGTGTGCCTGACGCTGGGGCTGGCGGGCGT<br>GATGGGTCTGGTCACGGATGTGGCGGCCGCCTGGAGCGGCAAGACCTCCGCCGCCGTGTTGATGGGCAG<br>CGGCGCGATCGTGGCGGTGTGGATGACGTTGATCCTGTTCCAACTGGTGCAGGGTAAAAACAGCGGGGT<br>GGTATTGTTGCAATATTACCTTGGCATGCTCGGTCTGTGTTGCCTCGGCGCCGCGGCGGCGTGGGCGGCG<br>GGCATGACCGGCATCGCCACCGGCGCCCTGCTGCTGGGGGGCGTGGTTGGCGGTGCGCTGTTCAGCAAC<br>CGGGCGGCGTTCTACCTGTATGTCGCCTATTTCCGCACGCGTCGGCGTGTGTTTATCAAACGCCGTTGGC<br>AGAGGGAAGATCTGCGCAACACCCGAGACTACAAAGACCATGACGGTGATTATAAAGATCATGATATC<br>GATTACAAGGATGACGATGACAAGTAAACCG
pSC3457	CTAGTGCTAGCGAATTCGAGCTCAGAGGACGTTAAAATGAAAAAGACAGCTATCGCGATTGCAGTGGCA<br>CTGGCTGGTTTCGCTACCGTAGCGCAGGCCGCTCCGAAATCTAGAAAAACTGCTTTTTCTACGCCGTTCG<br>GTTCTCCCGAAGACGATTTGACCAACGCCGAGTTTGCCCGCCTGTTGGGCAATACCGGCGTACAAGAAT<br>TCTATCGCGATATTAATCTGCAAGCCTTGCAGGACTCGCTGGACTGCAGCCTCGCCTTTCATGGCGTGGG<br>AATTATTTTTCCGACGGGCAAAACTCATTTTTAGTCCGCCCGACGCAGCACACCGGTTACTCGAACGCT<br>TCGCAGGTTACCCACGTGGTCATCAGCAAAACGGTGGCTCATACCTCGCGCGTTGCCGCGACCAATACG<br>CTGTCGGAAGCGTTGAGCAAACCCAGCGTCAGCAAAGAGCTGGCCTCGGCCGCGCTGTCGTGTGGAACG<br>CTGCTGGTATCGGTCTTTTTACTGGCGTCGGGCAGCGTCGCCGTGCCATTTACCGGCGGTACCAGCTCAG<br>CGGTGGCTTATCTGGGCTATGCCGGCATGGCCGCCAGCGCGTTGCAGTGCGGTAATGGGCTGTACCGCG<br>TCAACAAGCTTTATGACGGGAAAGGCGATGAATTGGCTCAGCTCGATTCAGAGCAGTGGTATATCGCCA<br>CCAGTACCGTGCTCGATGTGATCTCGCTGGCCAGCGCCGGGGCTGCCCTGAAGGAAGCGACGATGACCT<br>ATCGTGCCATGCGCCGCATTTCGGCGCGCAAGGCGACGGAGTGGCTGAAAAGCATGCCCCGCAGCGAA<br>AGGAAGCGACTGACCGAAAACATCATTCGGGCGGAAAATCCGGGAATTTCCAACAACGTTTTGAAAGA<br>GATGGTGAAGAACGGCCTGTACCCGAAACGCTACCCGACGGAAGCGATCCAAAACGGGCTGCGCCAGC<br>AATTGCACTCGGCGCTCAATAACGCGTTGACGTTTGTCGGCAGCGGCATCAGCGGCACCCTGTCGGCGC<br>CGGTGAATGTGAAAACCACCGGGCAGTATTTTGTGGGCATTATGCAGAAATTGCCGCAGATGGTGAGGT<br>AGGGCCC
pSC3459	CTAGTGCTAGCGAATTCGAGCTCAGAGGACGTTAAAATGAAAAAGACAGCTATCGCGATTGCAGTGGCA<br>CTGGCTGGTTTCGCTACCGTAGCGCAGGCCGCTCCGAAATCTAGAGCAAAAGGTGCGAAGGAAATCGCC<br>CAAGAGATGGCGAACGCTGTAAATTCAAAAAGTAATTTCTTTGGCGGTTTTATCGAAGGGGCTATCTCCT<br>TTCCCGTCGATATAGGATACCTGGCATATGATTTTATCAATACAGATAACCGCTCTATAAATCGATATGA<br>CACAGAAAGAATGCTTCGCCTCATTAAAGCTGGACTTGCTAACCAACACTCACTGACCAAAATCGTTAA<br>ACTTGTCGTTGACGAATATCTGAAAAAAGTCGATGTAGATAAAGTCAAAAGATGGGTTGAAAAAGGATC<br>CGGGAAAATTGCAGGTAGGTTTGTGAGCAATCAGGTTCTGATGGTTAACTTAGGGGCGGTGCTTTCCGA<br>ACGGGTGGTTATTCGCCTCGCAACAGGTTATGCCCTAACGTCACTGCTGACCCTCGGAGCTATGAACTCA<br>AGAGCAATCCATACCTCACGCCAGTTACGACAGCGAAACCCTGAAATTTACGATAACTTAAGGCGTGCG<br>GGAAATTTGGATCTCTTATATTTCTGGTAGAACCCAAAACAAAGCCATTTGAACAAGCGATAGAGATTT<br>GGCGAAAAAACAGAGGCGAGTTTGACCGCATTACCGAGCTATTTTTGAAAAGGTGTCGAAATAGCCC

Primer	Sequence (5’-3’)	Description
pIT2 Fwd 1	CTGGATGGAAAACGGGAAAGGTTCCGTCCA	Forward primer for amplification of C-tailed DNA fragments during Tn-seq
olj376	GTGACTGGAGTTCAGACGTGTGCTCTTCCGATC<br>TGGGGGGGGGGGGGG	Reverse primer for amplification of C-tailed DNA fragments during Tn-seq
pIT2 Fwd 2	AATGATACGGCGACCACCGAGATCTACACTCT<br>TTCCCTACACGACGCTCTTCCGATCTGGTTCCG<br>TCCAGGACGCTACTTGTGTATAAGAGT	Forward primer for final amplification of Tn-seq fragments for sequencing. Used with NEBNext index primers

References for Appendix

Alcoforado Diniz J, Coulthurst SJ (2015) Intraspecies Competition in *Serratia marcescens* Is Mediated by Type VI-Secreted Rhs Effectors and a Conserved Effector-Associated Accessory Protein. *J Bacteriol* 197: 2350-2360

Baba T, Ara T, Hasegawa M, Takai Y, Okumura Y, Baba M, Datsenko KA, Tomita M, Wanner BL, Mori H (2006) Construction of *Escherichia coli* K-12 in-frame, single-gene knockout mutants: the Keio collection. *Mol Syst Biol* 2: 2006 0008

Babin BM, Bergkessel M, Sweredoski MJ, Moradian A, Hess S, Newman DK, Tirrell DA (2016) SutA is a bacterial transcription factor expressed during slow growth in *Pseudomonas aeruginosa*. *Proc Natl Acad Sci U S A* 113: E597-605

Blattner FR, Plunkett G, 3rd, Bloch CA, Perna NT, Burland V, Riley M, Collado-Vides J, Glasner JD, Rode CK, Mayhew GF *et al* (1997) The complete genome sequence of *Escherichia coli* K-12. *Science* 277: 1453-1462

Cherepanov PP, Wackernagel W (1995) Gene disruption in *Escherichia coli*: Tc^R and Km^R cassettes with the option of Flp-catalyzed excision of the antibiotic-resistance determinant. *Gene* 158: 9-14

Choi KH, Schweizer HP (2006a) mini-Tn*7* insertion in bacteria with secondary, non-*glmS*-linked *att*Tn*7* sites: example *Proteus mirabilis* HI4320. *Nat Protoc* 1: 170-178

Choi KH, Schweizer HP (2006b) mini-Tn*7* insertion in bacteria with single *att*Tn*7* sites: example *Pseudomonas aeruginosa*. *Nat Protoc* 1: 153-161

Cianfanelli FR, Alcoforado Diniz J, Guo M, De Cesare V, Trost M, Coulthurst SJ (2016) VgrG and PAAR Proteins Define Distinct Versions of a Functional Type VI Secretion System. *PLoS Pathog* 12: e1005735

Flyg C, Kenne K, Boman HG (1980) Insect pathogenic properties of *Serratia marcescens*: phage-resistant mutants with a decreased resistance to *Cecropia* immunity and a decreased virulence to Drosophila. *J Gen Microbiol* 120: 173-181

Fritsch MJ, Trunk K, Diniz JA, Guo M, Trost M, Coulthurst SJ (2013) Proteomic identification of novel secreted antibacterial toxins of the *Serratia marcescens* type VI secretion system. *Mol Cell Proteomics* 12: 2735-2749

Gallagher LA, Ramage E, Patrapuvich R, Weiss E, Brittnacher M, Manoil C (2013) Sequence-defined transposon mutant library of *Burkholderia thailandensis*. *MBio* 4: e00604-00613

Gerc AJ, Diepold A, Trunk K, Porter M, Rickman C, Armitage JP, Stanley-Wall NR, Coulthurst SJ (2015) Visualization of the *Serratia* Type VI Secretion System Reveals Unprovoked Attacks and Dynamic Assembly. *Cell Rep* 12: 2131-2142

Grinter NJ (1983) A broad-host-range cloning vector transposable to various replicons. *Gene* 21: 133-143

Guzman LM, Belin D, Carson MJ, Beckwith J (1995) Tight regulation, modulation, and high-level expression by vectors containing the arabinose P_{BAD} promoter. *J Bacteriol* 177: 4121-4130

Herrero M, de Lorenzo V, Timmis KN (1990) Transposon vectors containing non-antibiotic resistance selection markers for cloning and stable chromosomal insertion of foreign genes in gram-negative bacteria. *J Bacteriol* 172: 6557-6567

Jack RL, Buchanan G, Dubini A, Hatzixanthis K, Palmer T, Sargent F (2004) Coordinating assembly and export of complex bacterial proteins. *EMBO J* 23: 3962-3972

Jacobs MA, Alwood A, Thaipisuttikul I, Spencer D, Haugen E, Ernst S, Will O, Kaul R, Raymond C, Levy R *et al* (2003) Comprehensive transposon mutant library of *Pseudomonas aeruginosa*. *Proc Natl Acad Sci U S A* 100: 14339-14344

Kaniga K, Delor I, Cornelis GR (1991) A wide-host-range suicide vector for improving reverse genetics in gram-negative bacteria: inactivation of the *blaA* gene of *Yersinia enterocolitica*. *Gene* 109: 137-141

Mariano G, Trunk K, Williams DJ, Monlezun L, Strahl H, Pitt SJ, Coulthurst SJ (2019) A family of Type VI secretion system effector proteins that form ion-selective pores. *Nat Commun* 10: 5484

Meisner J, Goldberg JB (2016) The *Escherichia coli rhaSR-PrhaBAD* Inducible Promoter System Allows Tightly Controlled Gene Expression over a Wide Range in *Pseudomonas aeruginosa*. *Appl Environ Microbiol* 82: 6715-6727

Murdoch SL, Trunk K, English G, Fritsch MJ, Pourkarimi E, Coulthurst SJ (2011) The opportunistic pathogen *Serratia marcescens* utilizes type VI secretion to target bacterial competitors. *J Bacteriol* 193: 6057-6069

Shanks RM, Caiazza NC, Hinsa SM, Toutain CM, O'Toole GA (2006) *Saccharomyces cerevisiae*-based molecular tool kit for manipulation of genes from gram-negative bacteria. *Appl Environ Microbiol* 72: 5027-5036

Trunk K, Peltier J, Liu YC, Dill BD, Walker L, Gow NAR, Stark MJR, Quinn J, Strahl H, Trost M *et al* (2018) The type VI secretion system deploys antifungal effectors against microbial competitors. *Nat Microbiol* 3: 920-931
