# Supplementary figures and images for: A widely-occurring family of pore-forming effectors broadens the impact of the Serratia Type VI secretion system
Source: EMBO J. 2025 Oct 21;44(23):6892–918. doi: 10.1038/s44318-025-00587-x (PMC12669606; doi:10.1038/s44318-025-00587-x)

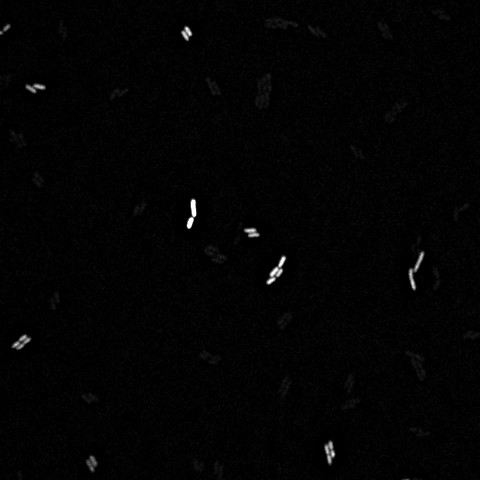

Supplement: Supplementary file 5 — Source data Fig. 1 [file 44318_2025_587_MOESM5_ESM.zip › Figure 1/1E/dSsp4 (mCherry) vs dSsp4Sip4 (GFP)/151019_AO03vsAO07-09_TL_01_30_R3D_D3D.dv_GFP_z04_t01.tiff]

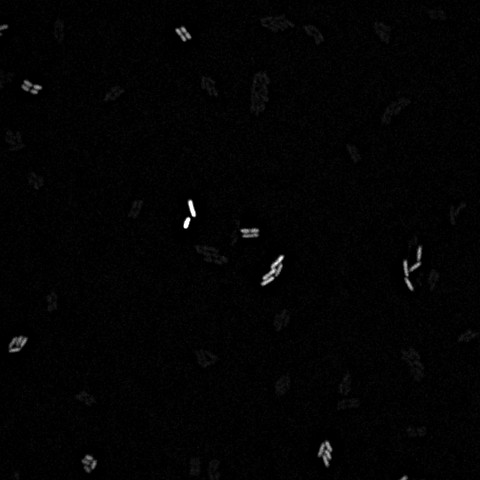

Supplement: Supplementary file 5 — Source data Fig. 1 [file 44318_2025_587_MOESM5_ESM.zip › Figure 1/1E/dSsp4 (mCherry) vs dSsp4Sip4 (GFP)/151019_AO03vsAO07-09_TL_01_30_R3D_D3D.dv_GFP_z04_t04.tiff]

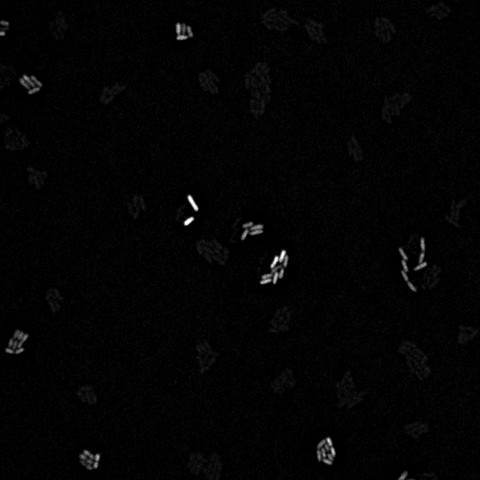

Supplement: Supplementary file 5 — Source data Fig. 1 [file 44318_2025_587_MOESM5_ESM.zip › Figure 1/1E/dSsp4 (mCherry) vs dSsp4Sip4 (GFP)/151019_AO03vsAO07-09_TL_01_30_R3D_D3D.dv_GFP_z04_t08.tiff]

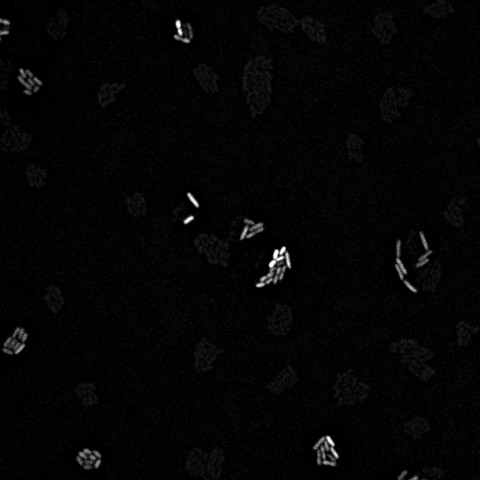

Supplement: Supplementary file 5 — Source data Fig. 1 [file 44318_2025_587_MOESM5_ESM.zip › Figure 1/1E/dSsp4 (mCherry) vs dSsp4Sip4 (GFP)/151019_AO03vsAO07-09_TL_01_30_R3D_D3D.dv_GFP_z04_t11.tiff]

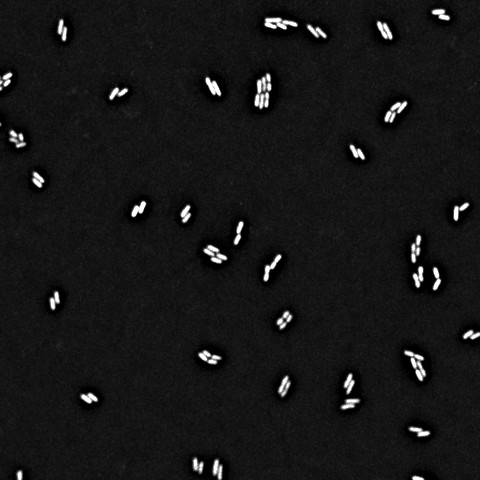

Supplement: Supplementary file 5 — Source data Fig. 1 [file 44318_2025_587_MOESM5_ESM.zip › Figure 1/1E/dSsp4 (mCherry) vs dSsp4Sip4 (GFP)/151019_AO03vsAO07-09_TL_01_30_R3D_D3D.dv_mCherry_z04_t01.tiff]

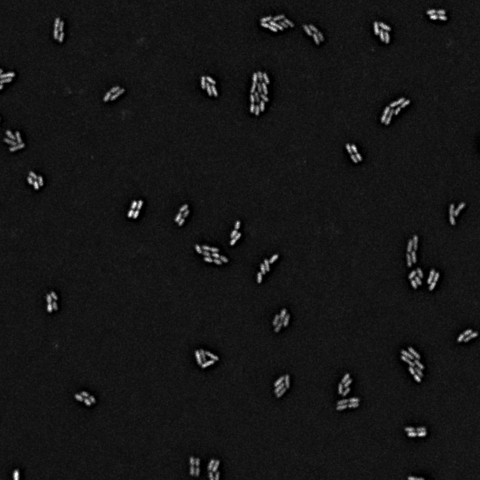

Supplement: Supplementary file 5 — Source data Fig. 1 [file 44318_2025_587_MOESM5_ESM.zip › Figure 1/1E/dSsp4 (mCherry) vs dSsp4Sip4 (GFP)/151019_AO03vsAO07-09_TL_01_30_R3D_D3D.dv_mCherry_z04_t04.tiff]

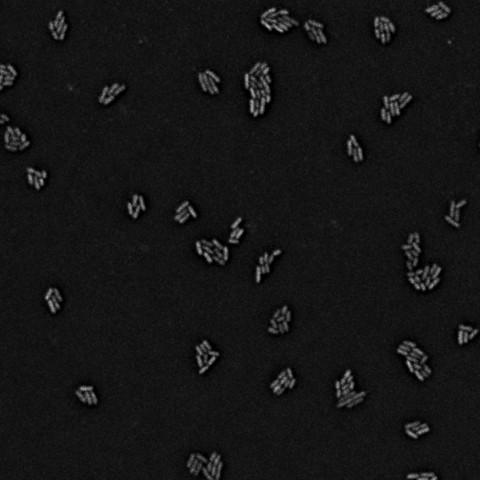

Supplement: Supplementary file 5 — Source data Fig. 1 [file 44318_2025_587_MOESM5_ESM.zip › Figure 1/1E/dSsp4 (mCherry) vs dSsp4Sip4 (GFP)/151019_AO03vsAO07-09_TL_01_30_R3D_D3D.dv_mCherry_z04_t08.tiff]

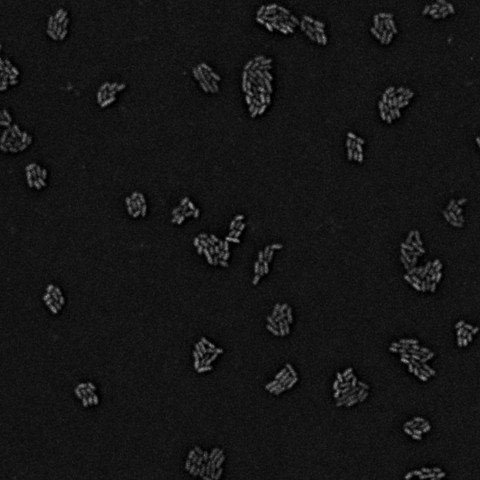

Supplement: Supplementary file 5 — Source data Fig. 1 [file 44318_2025_587_MOESM5_ESM.zip › Figure 1/1E/dSsp4 (mCherry) vs dSsp4Sip4 (GFP)/151019_AO03vsAO07-09_TL_01_30_R3D_D3D.dv_mCherry_z04_t11.tiff]

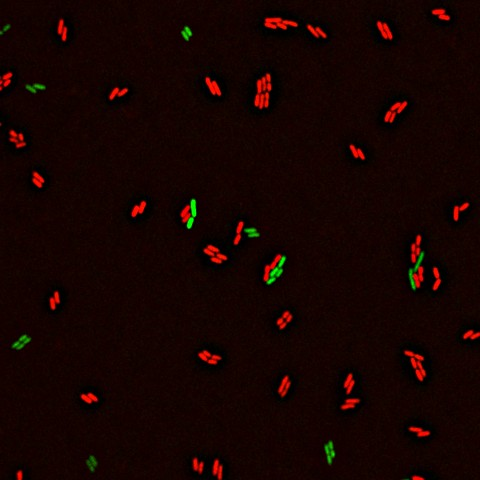

Supplement: Supplementary file 5 — Source data Fig. 1 [file 44318_2025_587_MOESM5_ESM.zip › Figure 1/1E/dSsp4 (mCherry) vs dSsp4Sip4 (GFP)/151019_AO03vsAO07-09_TL_01_30_R3D_D3D.dv_merged_z04_t01.tiff]

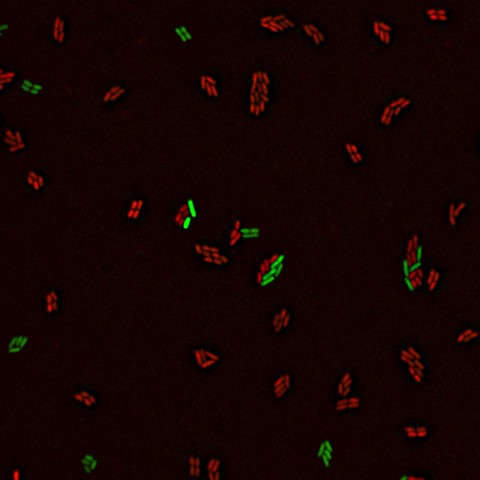

Supplement: Supplementary file 5 — Source data Fig. 1 [file 44318_2025_587_MOESM5_ESM.zip › Figure 1/1E/dSsp4 (mCherry) vs dSsp4Sip4 (GFP)/151019_AO03vsAO07-09_TL_01_30_R3D_D3D.dv_merged_z04_t04.tiff]

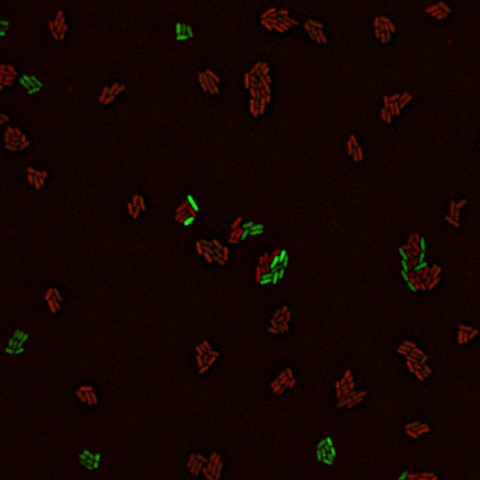

Supplement: Supplementary file 5 — Source data Fig. 1 [file 44318_2025_587_MOESM5_ESM.zip › Figure 1/1E/dSsp4 (mCherry) vs dSsp4Sip4 (GFP)/151019_AO03vsAO07-09_TL_01_30_R3D_D3D.dv_merged_z04_t08.tiff]

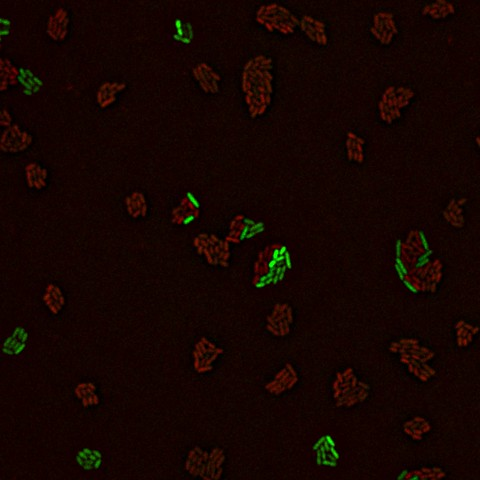

Supplement: Supplementary file 5 — Source data Fig. 1 [file 44318_2025_587_MOESM5_ESM.zip › Figure 1/1E/dSsp4 (mCherry) vs dSsp4Sip4 (GFP)/151019_AO03vsAO07-09_TL_01_30_R3D_D3D.dv_merged_z04_t11.tiff]

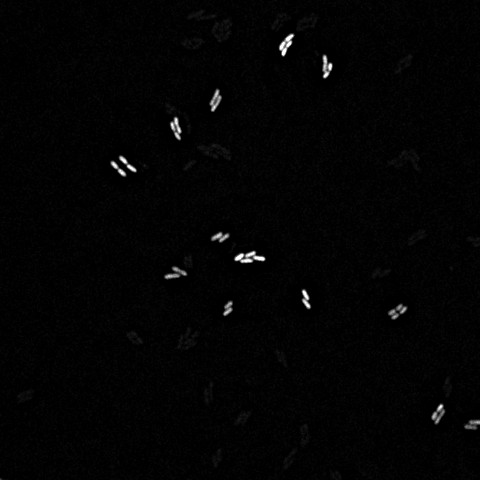

Supplement: Supplementary file 5 — Source data Fig. 1 [file 44318_2025_587_MOESM5_ESM.zip › Figure 1/1E/dTssE (mCherry) vs dSsp4dSip4 (GFP)/151019_AO03vsAO07-09_TL_01_12_R3D_D3D.dv_GFP_z04_t01.tiff]

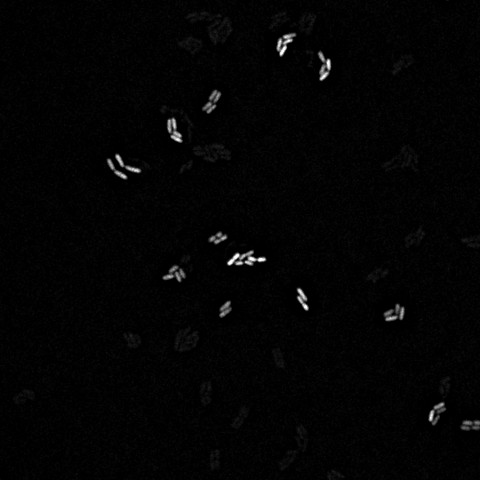

Supplement: Supplementary file 5 — Source data Fig. 1 [file 44318_2025_587_MOESM5_ESM.zip › Figure 1/1E/dTssE (mCherry) vs dSsp4dSip4 (GFP)/151019_AO03vsAO07-09_TL_01_12_R3D_D3D.dv_GFP_z04_t04.tiff]

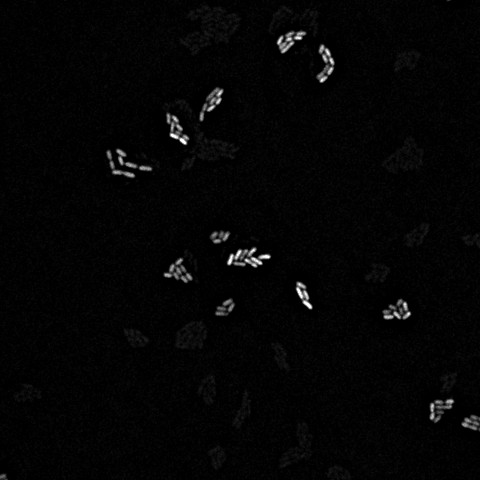

Supplement: Supplementary file 5 — Source data Fig. 1 [file 44318_2025_587_MOESM5_ESM.zip › Figure 1/1E/dTssE (mCherry) vs dSsp4dSip4 (GFP)/151019_AO03vsAO07-09_TL_01_12_R3D_D3D.dv_GFP_z04_t08.tiff]

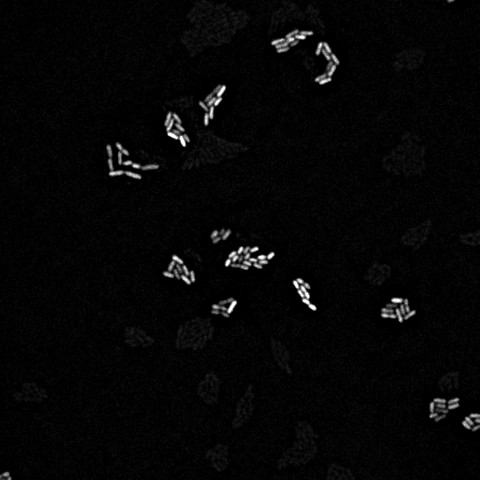

Supplement: Supplementary file 5 — Source data Fig. 1 [file 44318_2025_587_MOESM5_ESM.zip › Figure 1/1E/dTssE (mCherry) vs dSsp4dSip4 (GFP)/151019_AO03vsAO07-09_TL_01_12_R3D_D3D.dv_GFP_z04_t11.tiff]

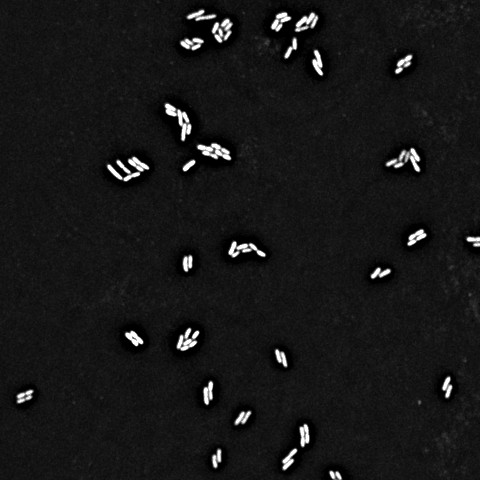

Supplement: Supplementary file 5 — Source data Fig. 1 [file 44318_2025_587_MOESM5_ESM.zip › Figure 1/1E/dTssE (mCherry) vs dSsp4dSip4 (GFP)/151019_AO03vsAO07-09_TL_01_12_R3D_D3D.dv_mCherry_z04_t01.tiff]

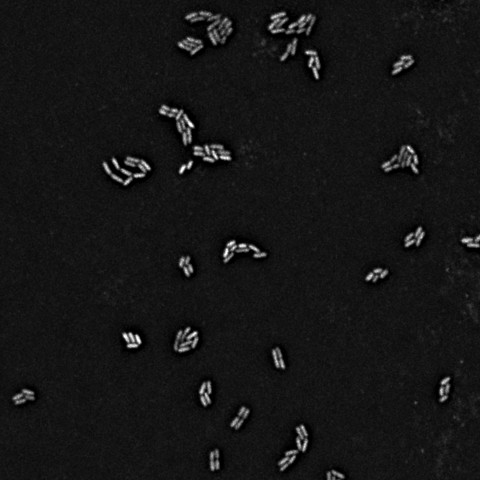

Supplement: Supplementary file 5 — Source data Fig. 1 [file 44318_2025_587_MOESM5_ESM.zip › Figure 1/1E/dTssE (mCherry) vs dSsp4dSip4 (GFP)/151019_AO03vsAO07-09_TL_01_12_R3D_D3D.dv_mCherry_z04_t04.tiff]

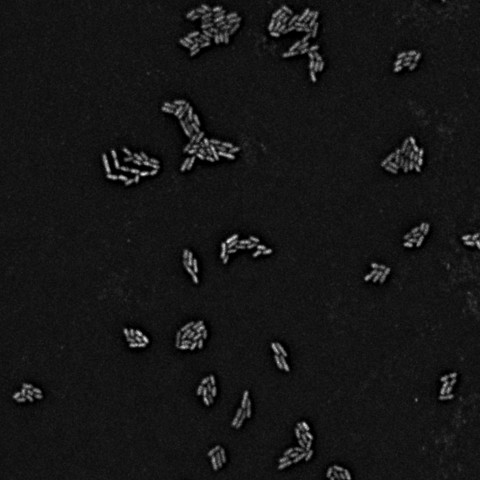

Supplement: Supplementary file 5 — Source data Fig. 1 [file 44318_2025_587_MOESM5_ESM.zip › Figure 1/1E/dTssE (mCherry) vs dSsp4dSip4 (GFP)/151019_AO03vsAO07-09_TL_01_12_R3D_D3D.dv_mCherry_z04_t08.tiff]

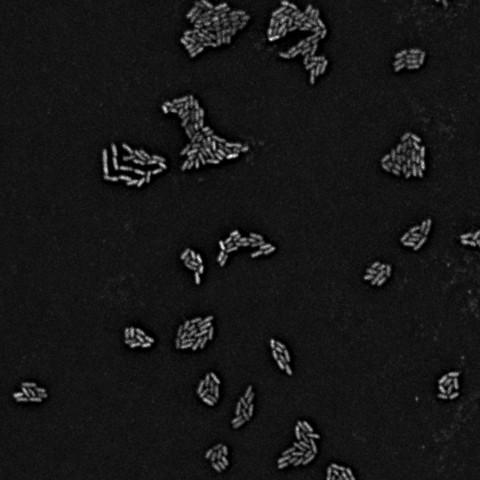

Supplement: Supplementary file 5 — Source data Fig. 1 [file 44318_2025_587_MOESM5_ESM.zip › Figure 1/1E/dTssE (mCherry) vs dSsp4dSip4 (GFP)/151019_AO03vsAO07-09_TL_01_12_R3D_D3D.dv_mCherry_z04_t11.tiff]

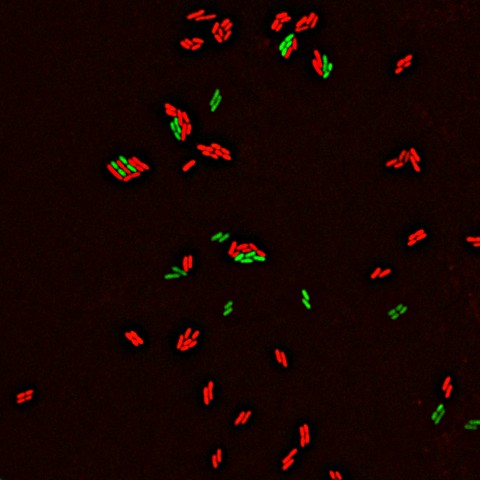

Supplement: Supplementary file 5 — Source data Fig. 1 [file 44318_2025_587_MOESM5_ESM.zip › Figure 1/1E/dTssE (mCherry) vs dSsp4dSip4 (GFP)/151019_AO03vsAO07-09_TL_01_12_R3D_D3D.dv_merged_z04_t01.tiff]

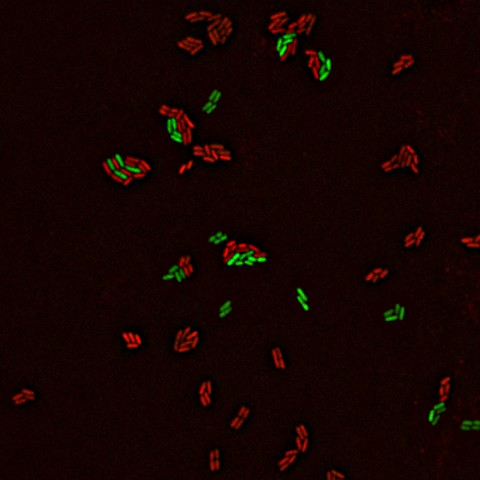

Supplement: Supplementary file 5 — Source data Fig. 1 [file 44318_2025_587_MOESM5_ESM.zip › Figure 1/1E/dTssE (mCherry) vs dSsp4dSip4 (GFP)/151019_AO03vsAO07-09_TL_01_12_R3D_D3D.dv_merged_z04_t04.tiff]

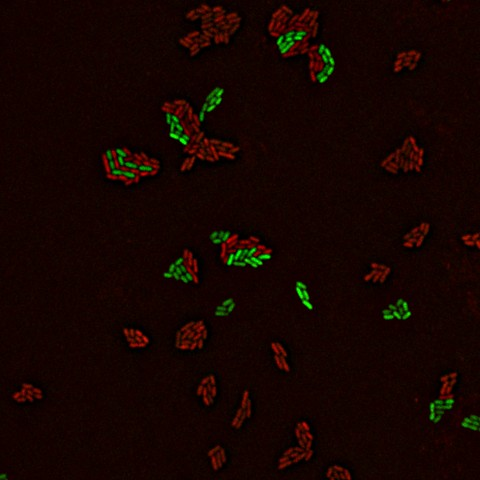

Supplement: Supplementary file 5 — Source data Fig. 1 [file 44318_2025_587_MOESM5_ESM.zip › Figure 1/1E/dTssE (mCherry) vs dSsp4dSip4 (GFP)/151019_AO03vsAO07-09_TL_01_12_R3D_D3D.dv_merged_z04_t08.tiff]

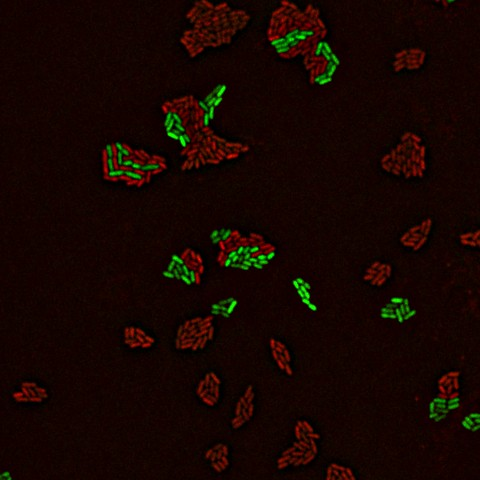

Supplement: Supplementary file 5 — Source data Fig. 1 [file 44318_2025_587_MOESM5_ESM.zip › Figure 1/1E/dTssE (mCherry) vs dSsp4dSip4 (GFP)/151019_AO03vsAO07-09_TL_01_12_R3D_D3D.dv_merged_z04_t11.tiff]

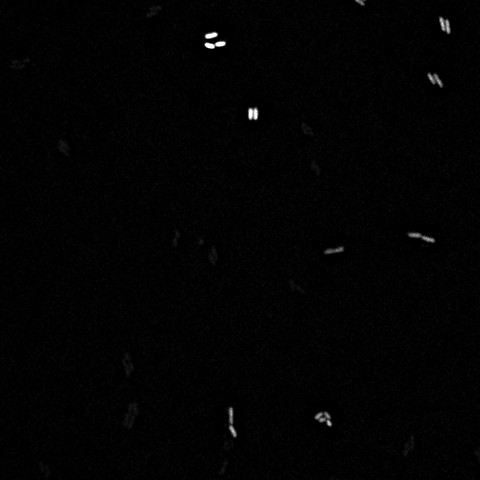

Supplement: Supplementary file 5 — Source data Fig. 1 [file 44318_2025_587_MOESM5_ESM.zip › Figure 1/1E/WT (mCherry) vs dSsp4dSip4 (GFP)/151019_AO03vsAO07-09_TL_01_01_R3D_D3D.dv_GFP_z04_t01.tiff]

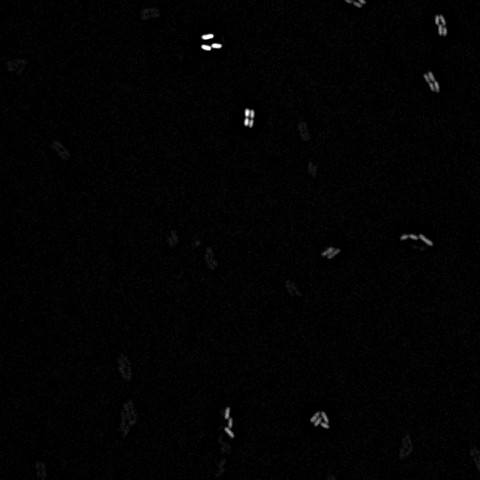

Supplement: Supplementary file 5 — Source data Fig. 1 [file 44318_2025_587_MOESM5_ESM.zip › Figure 1/1E/WT (mCherry) vs dSsp4dSip4 (GFP)/151019_AO03vsAO07-09_TL_01_01_R3D_D3D.dv_GFP_z04_t04.tiff]

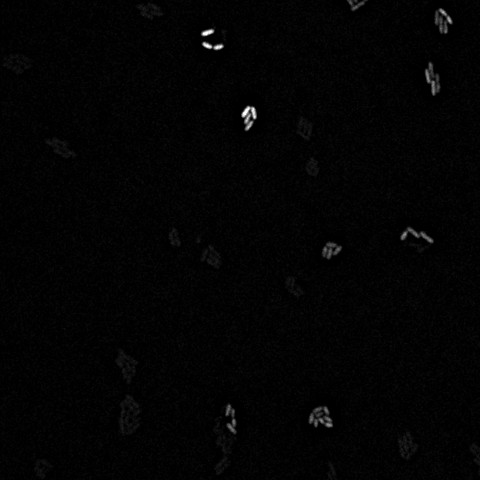

Supplement: Supplementary file 5 — Source data Fig. 1 [file 44318_2025_587_MOESM5_ESM.zip › Figure 1/1E/WT (mCherry) vs dSsp4dSip4 (GFP)/151019_AO03vsAO07-09_TL_01_01_R3D_D3D.dv_GFP_z04_t08.tiff]

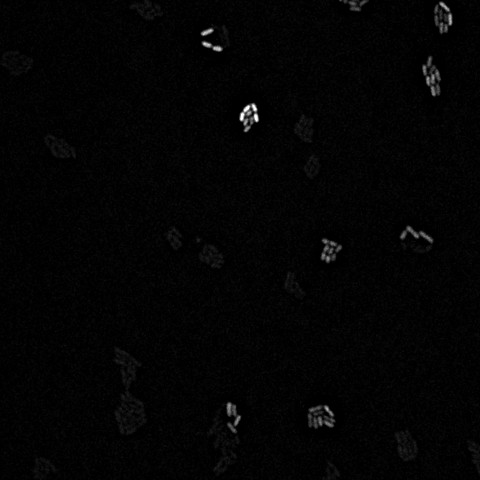

Supplement: Supplementary file 5 — Source data Fig. 1 [file 44318_2025_587_MOESM5_ESM.zip › Figure 1/1E/WT (mCherry) vs dSsp4dSip4 (GFP)/151019_AO03vsAO07-09_TL_01_01_R3D_D3D.dv_GFP_z04_t11.tiff]

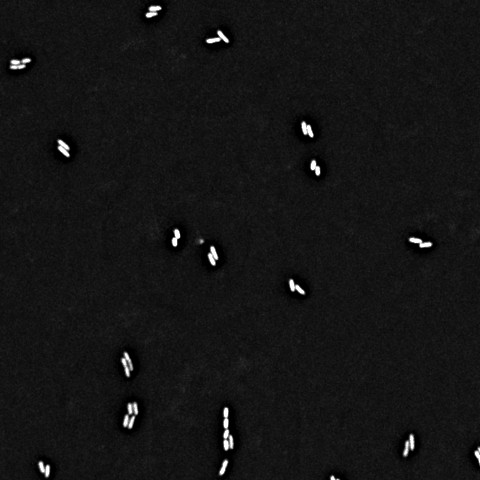

Supplement: Supplementary file 5 — Source data Fig. 1 [file 44318_2025_587_MOESM5_ESM.zip › Figure 1/1E/WT (mCherry) vs dSsp4dSip4 (GFP)/151019_AO03vsAO07-09_TL_01_01_R3D_D3D.dv_mCherry_z04_t01.tiff]

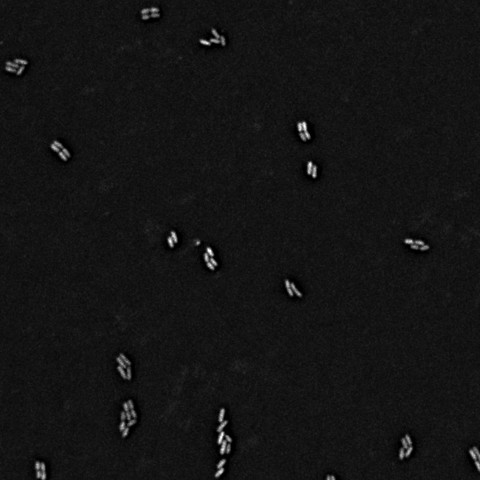

Supplement: Supplementary file 5 — Source data Fig. 1 [file 44318_2025_587_MOESM5_ESM.zip › Figure 1/1E/WT (mCherry) vs dSsp4dSip4 (GFP)/151019_AO03vsAO07-09_TL_01_01_R3D_D3D.dv_mCherry_z04_t04.tiff]

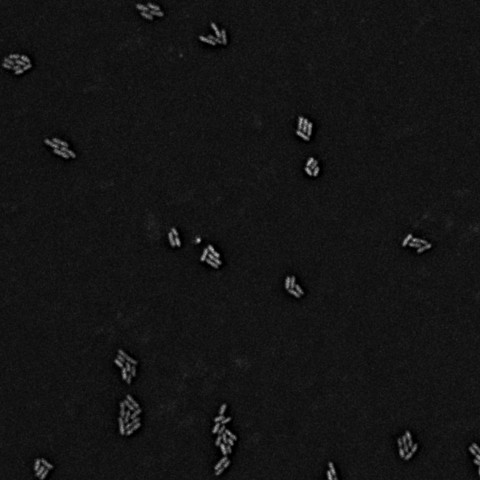

Supplement: Supplementary file 5 — Source data Fig. 1 [file 44318_2025_587_MOESM5_ESM.zip › Figure 1/1E/WT (mCherry) vs dSsp4dSip4 (GFP)/151019_AO03vsAO07-09_TL_01_01_R3D_D3D.dv_mCherry_z04_t08.tiff]

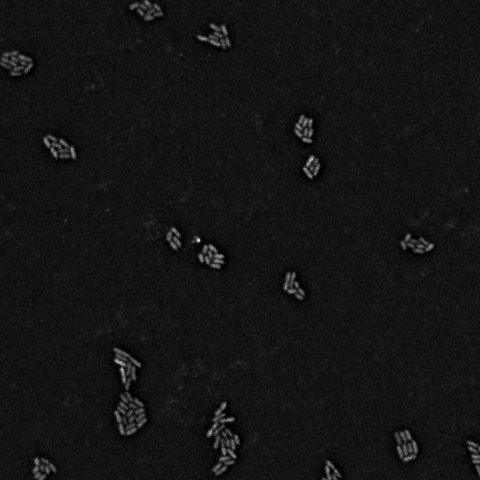

Supplement: Supplementary file 5 — Source data Fig. 1 [file 44318_2025_587_MOESM5_ESM.zip › Figure 1/1E/WT (mCherry) vs dSsp4dSip4 (GFP)/151019_AO03vsAO07-09_TL_01_01_R3D_D3D.dv_mCherry_z04_t11.tiff]

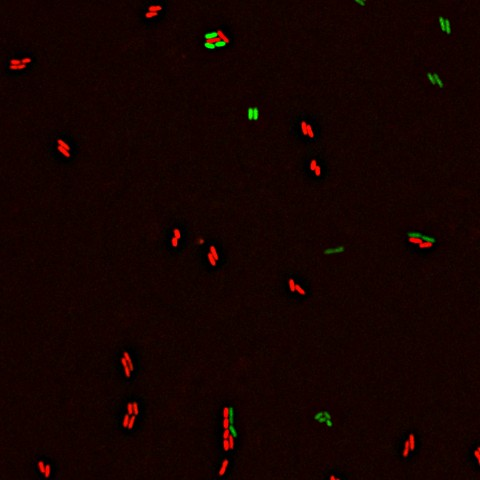

Supplement: Supplementary file 5 — Source data Fig. 1 [file 44318_2025_587_MOESM5_ESM.zip › Figure 1/1E/WT (mCherry) vs dSsp4dSip4 (GFP)/151019_AO03vsAO07-09_TL_01_01_R3D_D3D.dv_merged_z04_t01.tiff]

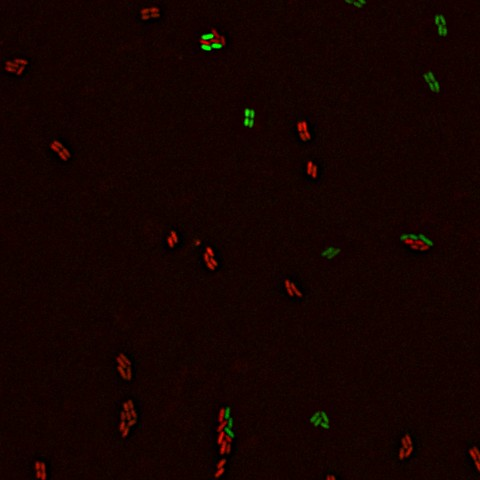

Supplement: Supplementary file 5 — Source data Fig. 1 [file 44318_2025_587_MOESM5_ESM.zip › Figure 1/1E/WT (mCherry) vs dSsp4dSip4 (GFP)/151019_AO03vsAO07-09_TL_01_01_R3D_D3D.dv_merged_z04_t04.tiff]

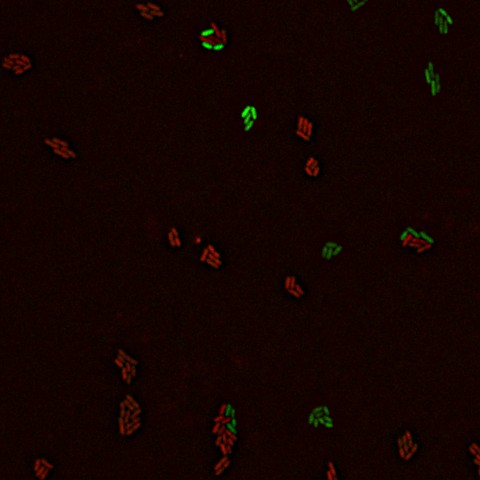

Supplement: Supplementary file 5 — Source data Fig. 1 [file 44318_2025_587_MOESM5_ESM.zip › Figure 1/1E/WT (mCherry) vs dSsp4dSip4 (GFP)/151019_AO03vsAO07-09_TL_01_01_R3D_D3D.dv_merged_z04_t08.tiff]

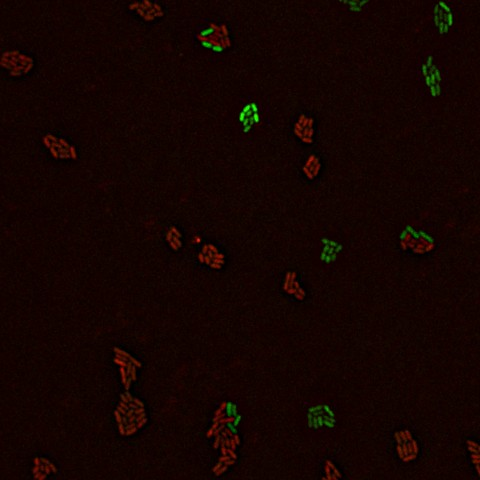

Supplement: Supplementary file 5 — Source data Fig. 1 [file 44318_2025_587_MOESM5_ESM.zip › Figure 1/1E/WT (mCherry) vs dSsp4dSip4 (GFP)/151019_AO03vsAO07-09_TL_01_01_R3D_D3D.dv_merged_z04_t11.tiff]

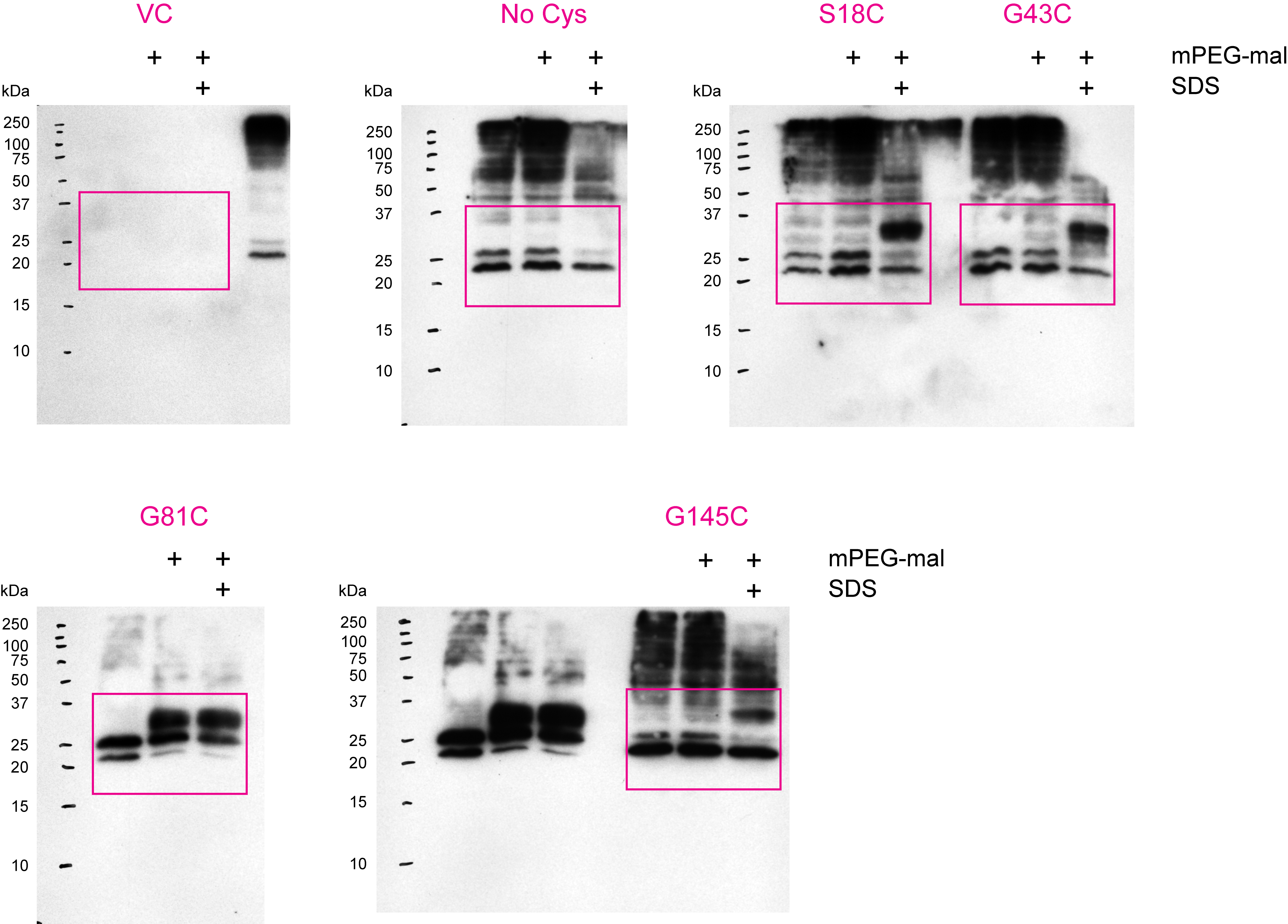

Supplement: Supplementary file 5 — Source data Fig. 1 [file 44318_2025_587_MOESM5_ESM.zip › Figure 1/1G/Annotated blots.png]

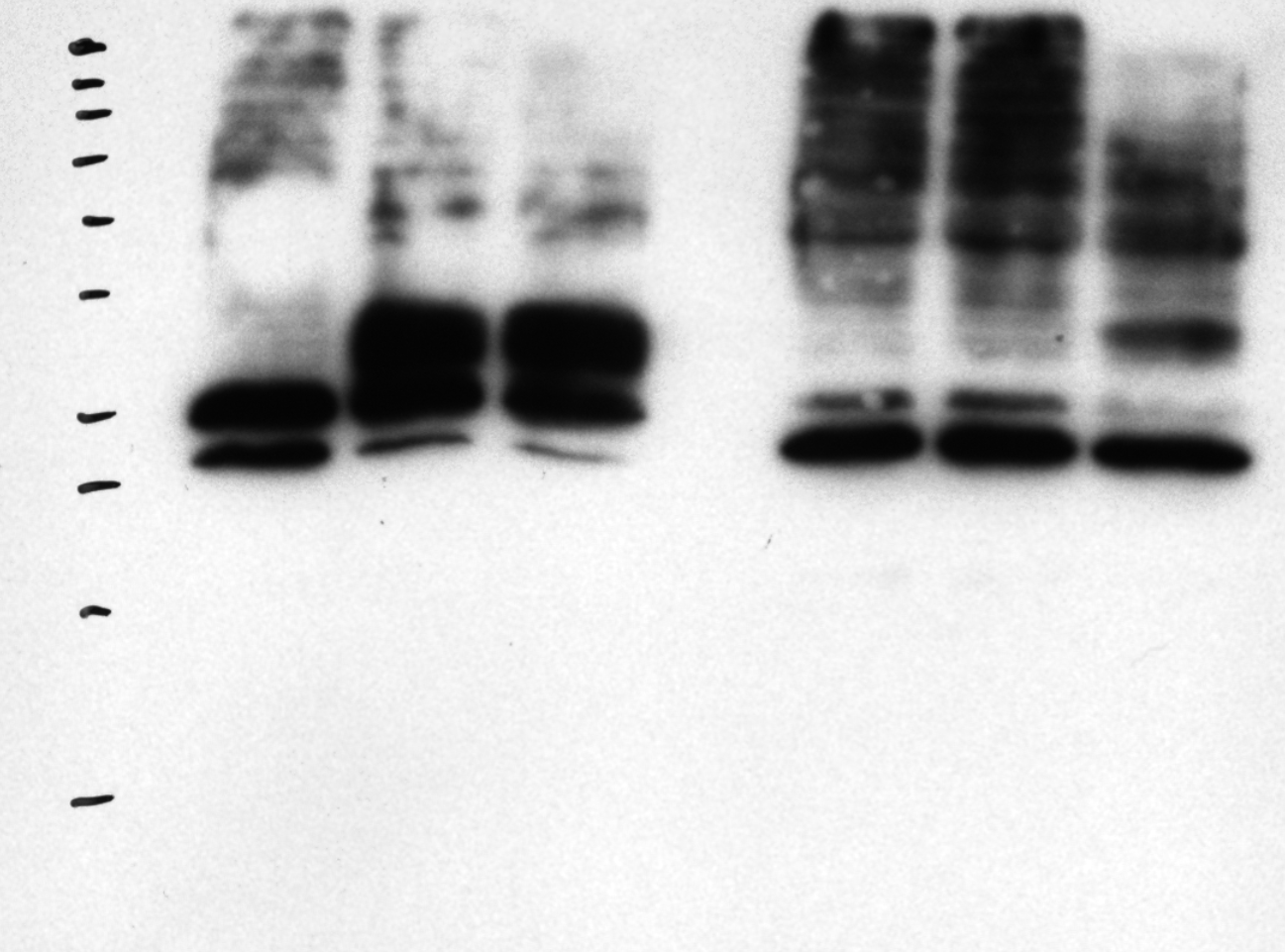

Supplement: Supplementary file 5 — Source data Fig. 1 [file 44318_2025_587_MOESM5_ESM.zip › Figure 1/1G/G145C blot (anti-FLAG).tif]

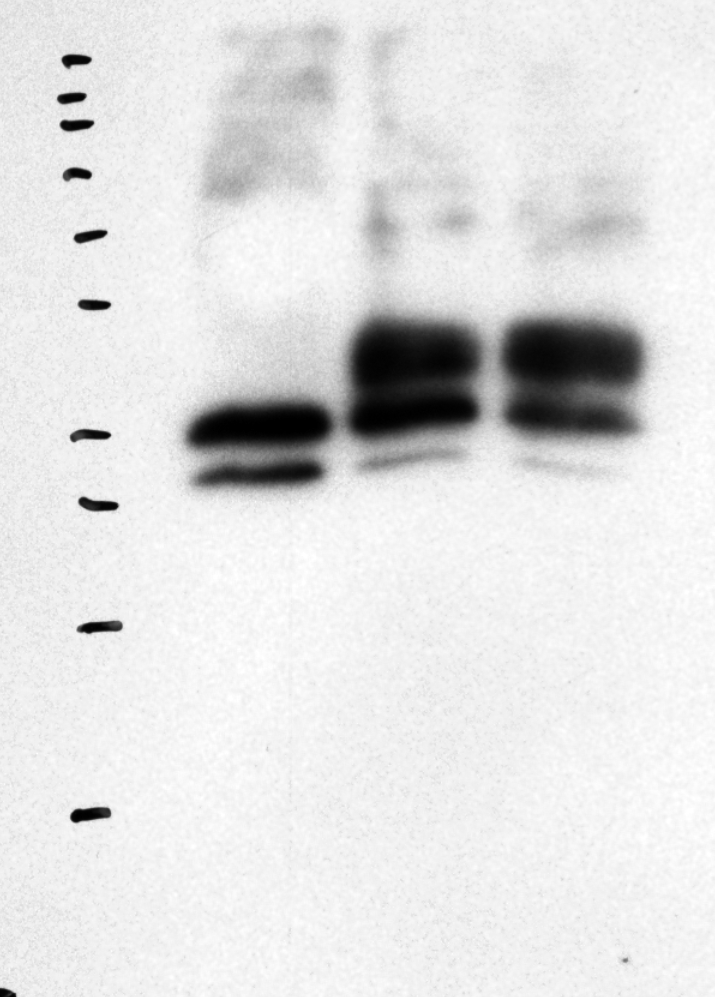

Supplement: Supplementary file 5 — Source data Fig. 1 [file 44318_2025_587_MOESM5_ESM.zip › Figure 1/1G/G81C blot (anti-FLAG).tif]

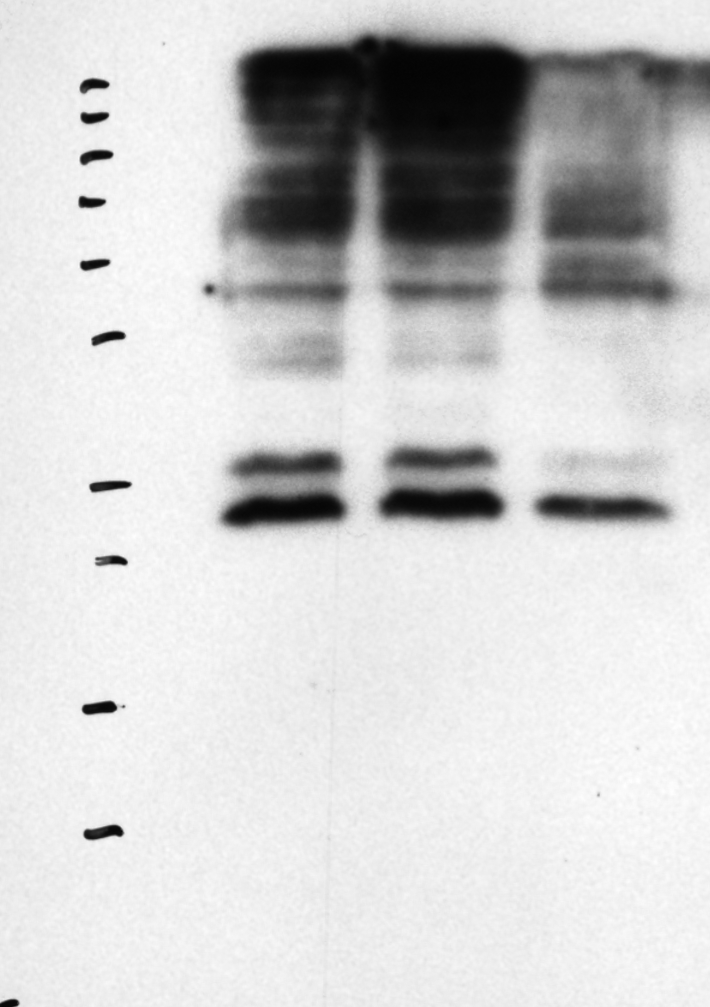

Supplement: Supplementary file 5 — Source data Fig. 1 [file 44318_2025_587_MOESM5_ESM.zip › Figure 1/1G/No Cys blot (anti-FLAG).tif]

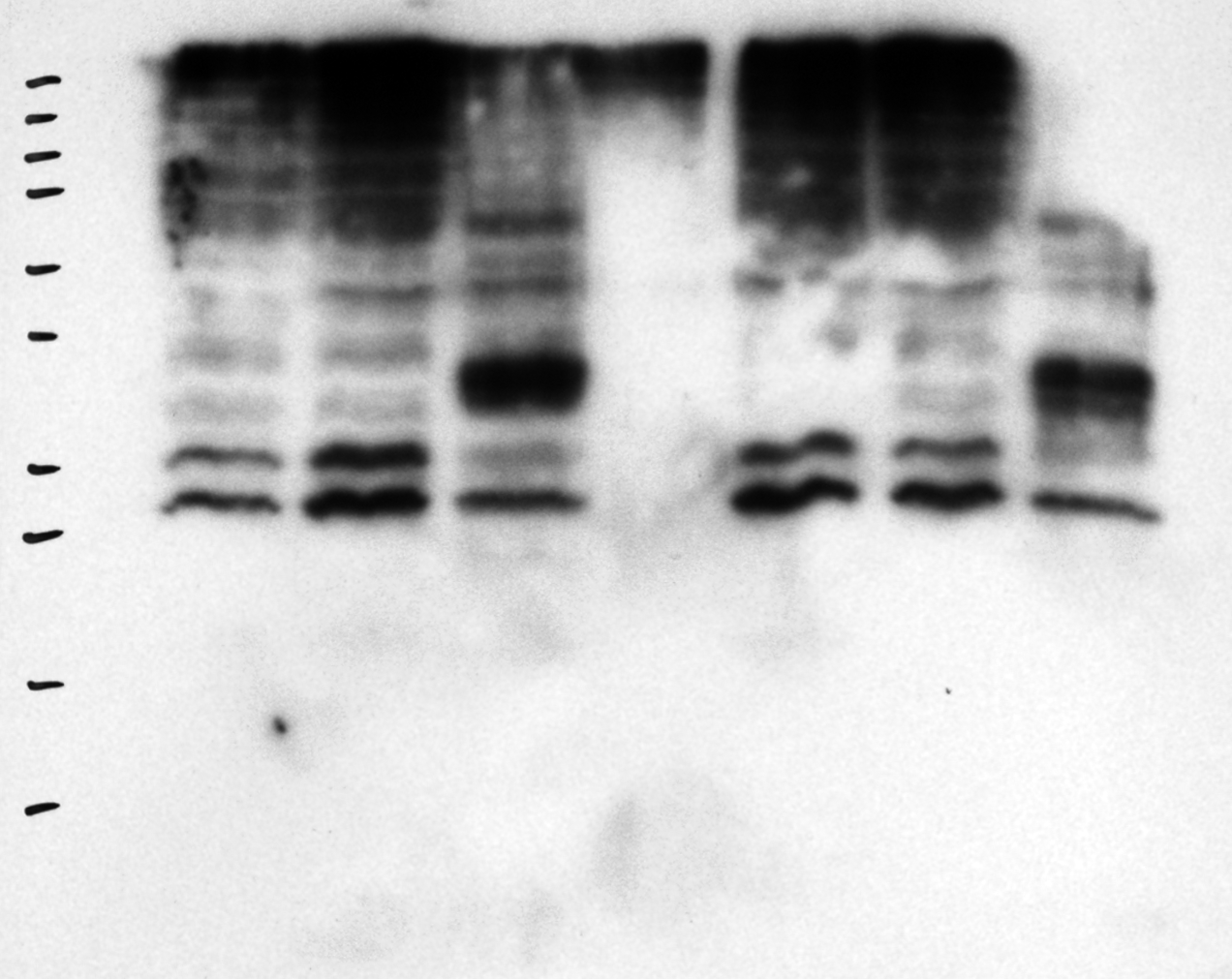

Supplement: Supplementary file 5 — Source data Fig. 1 [file 44318_2025_587_MOESM5_ESM.zip › Figure 1/1G/S18C & G43C blot (anti-FLAG).tif]

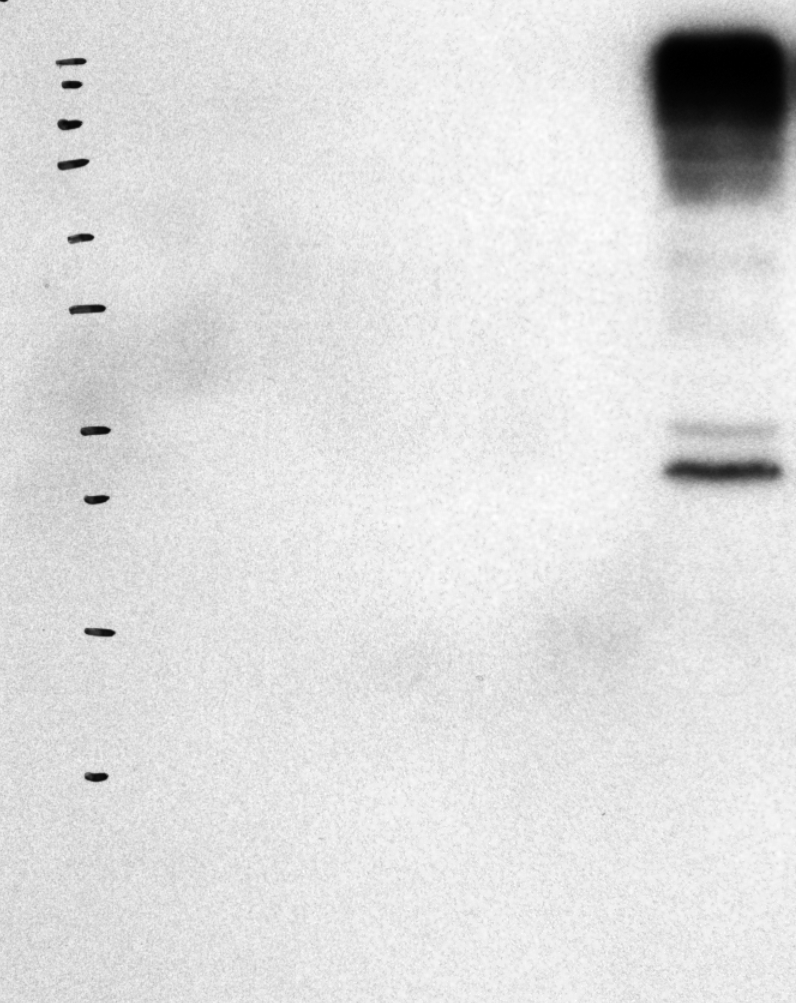

Supplement: Supplementary file 5 — Source data Fig. 1 [file 44318_2025_587_MOESM5_ESM.zip › Figure 1/1G/VC blot (anti-FLAG).tif]
