# Supplementary material for: A widely-occurring family of pore-forming effectors broadens the impact of the Serratia Type VI secretion system
Source: EMBO J. 2025 Oct 21;44(23):6892–918. doi: 10.1038/s44318-025-00587-x (PMC12669606; doi:10.1038/s44318-025-00587-x)
Supplement: Supplementary file 13 — Expanded View Figures [file 44318_2025_587_MOESM13_ESM.pdf]

Expanded View Figures

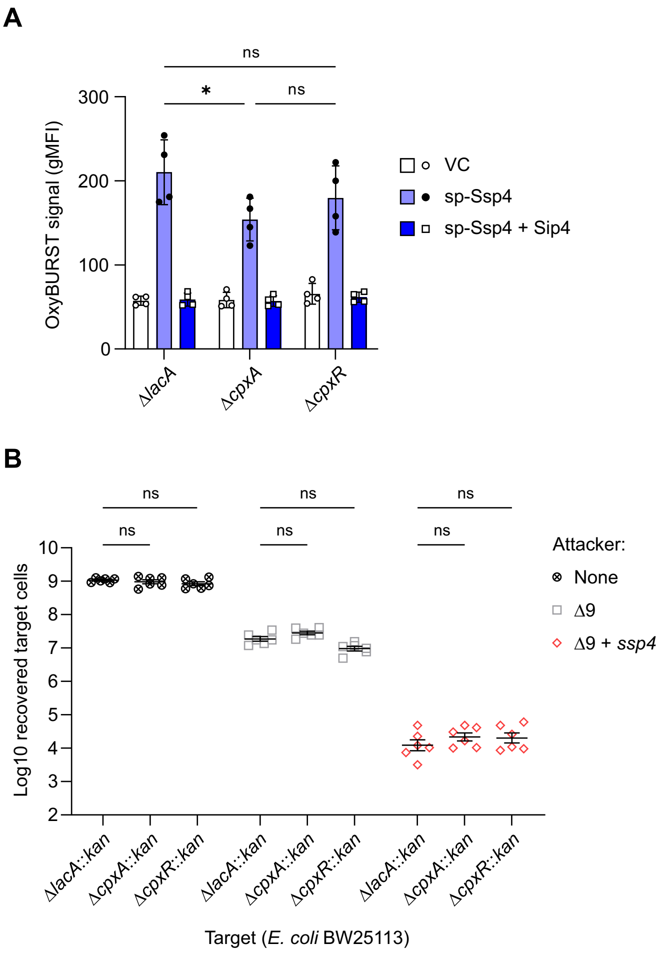

Figure EV1. Loss of the Cpx system in *E. coli* has little or no impact on susceptibility to Ssp4 intoxication.

(**A**) Quantification of OxyBURST Green intensity from strains of *E. coli* BW25113 (Δ*lacA*, Δ*cpxA*, Δ*cpxR*) carrying plasmids directing expression of sp-Ssp4 or sp-Ssp6, following 3 h induction. (**B**) Recovery of *E. coli* BW25112 carrying control (Δ*lacA*::*kan*) or Cpx (Δ*cpxA*::*kan*, Δ*cpxR*::*kan*) gene deletions, following co-culture with attacking strains of *S. marcescens* Db10 lacking known anti-bacterial effectors (Δ9) or delivering only Ssp4 (Δ9 + *ssp4*). None, no-attacker; *kan*, kanamycin-resistance gene replacing the deleted gene and providing selection for target cells. Data were presented as mean ± SEM with individual data points overlaid ($n = 4$ or $n = 6$ biological replicates in panels (**A**, **B**), respectively); *$P < 0.05$, ns not significant, one-way ANOVA with Tukey's test; for clarity, only selected comparisons are displayed. P values from left to right (**A**) $P = 0.0189$, $P = 0.5232$, $P = 0.7222$; (**B**) $P > 0.9999$, $P = 0.9978$, $P = 0.9339$, $P = 0.4848$, $P = 0.6823$, $P = 0.8057$. Source data are available online for this figure.

 © The Author(s)

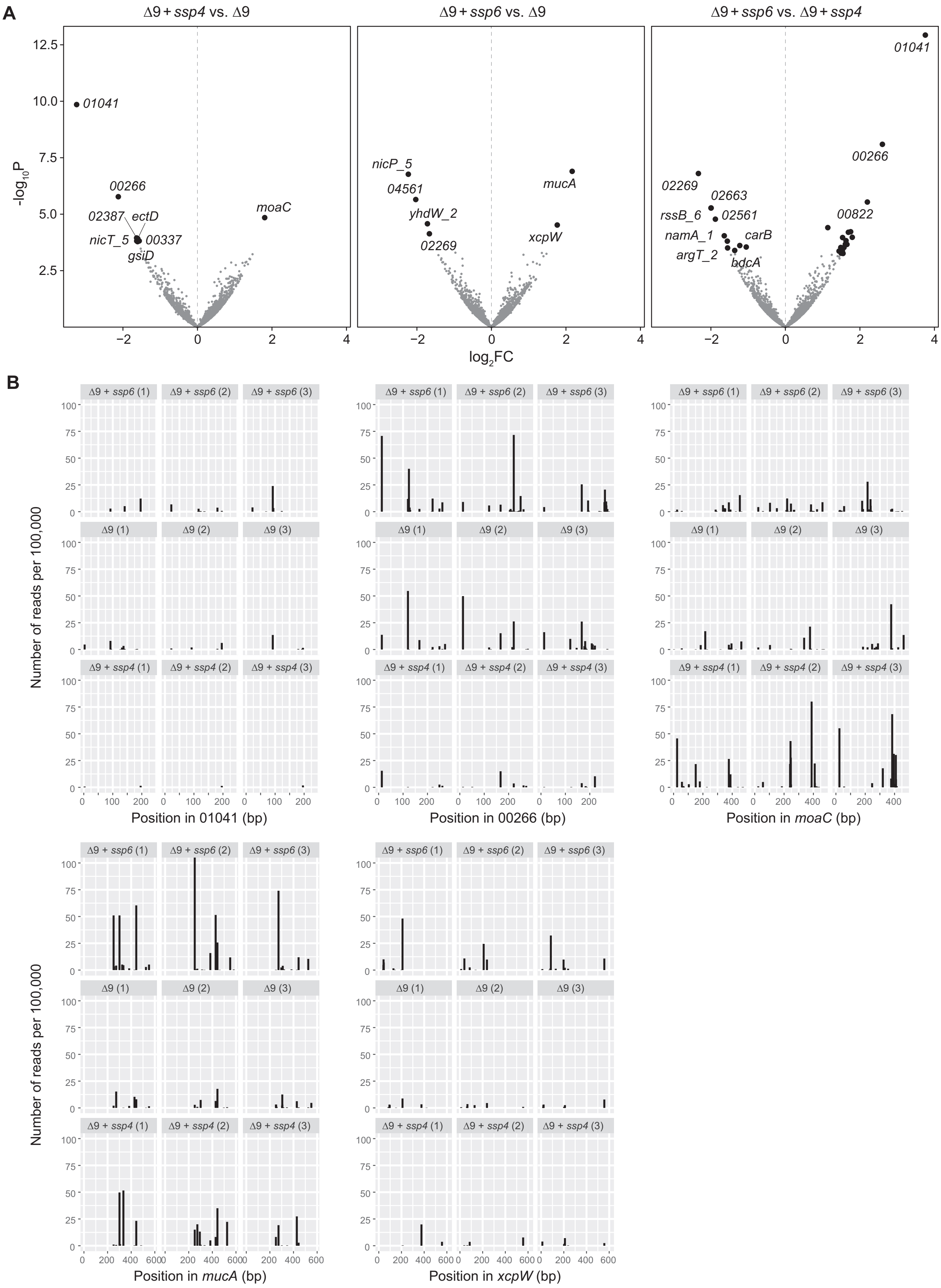
A
Δ9 + ssp4 vs. Δ9
Δ9 + ssp6 vs. Δ9
Δ9 + ssp6 vs. Δ9 + ssp4
-log10P
log2FC
01041
00266
02387
ectD
nicT_5
00337
gsiD
moaC
nicP_5
04561
yhdW_2
02269
mucA
xcpW
02663
rssB_6
02561
namA_1
carB
argT_2
bdcA
00822
B
Δ9 + ssp6 (1)
Δ9 + ssp6 (2)
Δ9 + ssp6 (3)
Δ9 (1)
Δ9 (2)
Δ9 (3)
Δ9 + ssp4 (1)
Δ9 + ssp4 (2)
Δ9 + ssp4 (3)
Number of reads per 100,000
Position in 01041 (bp)
Position in 00266 (bp)
Position in moaC (bp)
Position in mucA (bp)
Position in xcpW (bp)

Figure EV2. All three Tn-seq pairwise comparisons and individual insertion sites in genes of interest.

(**A**) Volcano plots summarising the change in recovery of *P. fluorescens* 55 transposon insertion mutants between control (Δ9) and Ssp4-delivering attackers (left), between control and Ssp6-delivering attackers (middle), and between Ssp4- and Ssp6-delivering attackers (right) on a per gene basis. Log2 fold change in normalised read count is plotted against −log10 *P* value and genes significantly altered between condition (FDR <0.05, EdgeR's quasi-likelihood *F*-test (QLF test) with Benjamini–Hochberg correction) are highlighted as black dots, with all (left, middle) or selected (right) gene annotations. (**B**) Position and number of sequencing reads for individual transposon insertion sites across five genes of interest in each replicate for each attacking strain.

 © The Author(s)

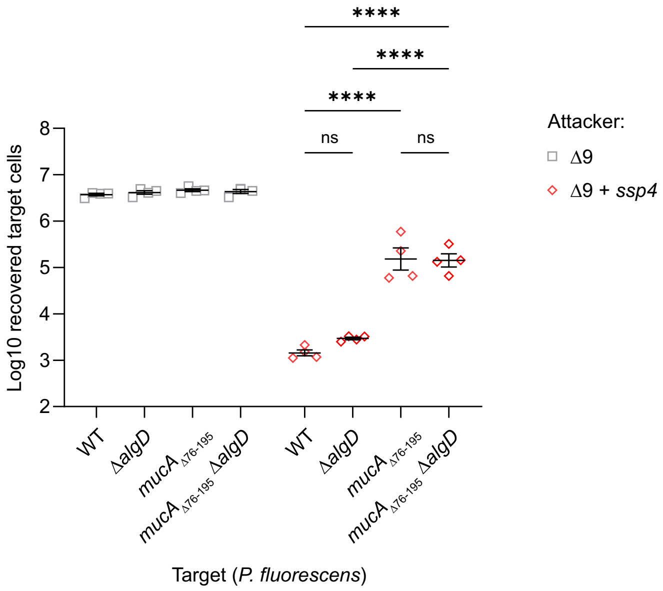

Figure EV3. Deletion of an alginate biosynthesis gene does not affect the resistance of the *P. fluorescens mucA*$_{\Delta 76–195}$ mutant to T6SS attacks.

Recovery of wild type (WT) or defined deletion mutants (Δ*algD, mucA*$_{\Delta 76–195}$, or *mucA*$_{\Delta 76–195}$ Δ*algD*) of *P. fluorescens* 55, following co-culture with attacking strains of *S. marcescens* Db10 as indicated. Data are presented as mean ± SEM with individual data points overlaid ($n = 4$ biological replicates); ****$P < 0.0001$, ns not significant; one-way ANOVA with Tukey's test; for clarity, only selected comparisons are displayed. P values from left to right $P = 0.4335$, $P < 0.0001$, $P < 0.0001$, $P < 0.0001$ and $P > 0.9999$. Source data are available online for this figure.

© The Author(s)
